# Blood transcriptional biomarkers for active pulmonary tuberculosis in a high-burden setting: a prospective, observational, diagnostic accuracy study

**DOI:** 10.1016/S2213-2600(19)30469-2

**Published:** 2020-04

**Authors:** Carolin T Turner, Rishi K Gupta, Evdokia Tsaliki, Jennifer K Roe, Prasenjit Mondal, Georgina R Nyawo, Zaida Palmer, Robert F Miller, Byron WP Reeve, Grant Theron, Mahdad Noursadeghi

**Affiliations:** a Division of Infection and Immunity, University College London, London, UK; b Institute for Global Health, University College London, London, UK; c DST-NRF Centre of Excellence for Biomedical Tuberculosis Research; South African Medical Research Council Centre for Tuberculosis Research; and Division of Molecular Biology and Human Genetics, Faculty of Medicine and Health Sciences, Stellenbosch University, Cape Town, South Africa

## Abstract

**Background:**

Blood transcriptional signatures are candidates for non-sputum triage or confirmatory tests of tuberculosis. Prospective head-to-head comparisons of their diagnostic accuracy in real-world settings are necessary to assess their clinical use. We aimed to compare the diagnostic accuracy of candidate transcriptional signatures identified by systematic review, in a setting with a high burden of tuberculosis and HIV.

**Methods:**

We did a prospective observational study nested within a diagnostic accuracy study of sputum Xpert MTB/RIF (Xpert) and Xpert MTB/RIF Ultra (Ultra) tests for pulmonary tuberculosis. We recruited consecutive symptomatic adults aged 18 years or older self-presenting to a tuberculosis clinic in Cape Town, South Africa. Participants provided blood for RNA sequencing, and sputum samples for liquid culture and molecular testing using Xpert and Ultra. We assessed the diagnostic accuracy of candidate blood transcriptional signatures for active tuberculosis (including those intended to distinguish active tuberculosis from other diseases) identified by systematic review, compared with culture or Xpert MTB/RIF positivity as the standard reference. In our primary analysis, patients with tuberculosis were defined as those with either a positive liquid culture or Xpert result. Patients with missing blood RNA or sputum results were excluded. Our primary objective was to benchmark the diagnostic accuracy of candidate transcriptional signatures against the WHO target product profile (TPP) for a tuberculosis triage test.

**Findings:**

Between Feb 12, 2016, and July 18, 2017, we obtained paired sputum and RNA sequencing data from 181 participants, 54 (30%) of whom had confirmed pulmonary tuberculosis. Of 27 eligible signatures identified by systematic review, four achieved the highest diagnostic accuracy with similar area under the receiver operating characteristic curves (Sweeney3: 90·6% [95% CI 85·6–95·6]; Kaforou25: 86·9% [80·9–92·9]; Roe3: 86·9% [80·3–93·5]; and BATF2: 86·8% [80·6–93·1]), independent of age, sex, HIV status, previous tuberculosis, or sputum smear result. At test thresholds that gave 70% specificity (the minimum WHO TPP specificity for a triage test), these four signatures achieved sensitivities between 83·3% (95% CI 71·3–91·0) and 90·7% (80·1–96·0). No signature met the optimum criteria, of 95% sensitivity and 80% specificity proposed by WHO for a triage test, or the minimum criteria (of 65% sensitivity and 98% specificity) for a confirmatory test, but all four correctly identified Ultra-positive, culture-negative patients.

**Interpretation:**

Selected blood transcriptional signatures met the minimum WHO benchmarks for a tuberculosis triage test but not for a confirmatory test. Further development of the signatures is warranted to investigate their possible effects on clinical and health economic outcomes as part of a triage strategy, or when used as add-on confirmatory test in conjunction with the highly sensitive Ultra test for *Mycobacterium tuberculosis* DNA.

**Funding:**

Royal Society Newton Advanced Fellowship, Wellcome Trust, National Institute of Health Research, and UK Medical Research Council.

## Introduction

Delays in diagnosis of active tuberculosis contribute to its high death toll and facilitate onward transmission of infection.^1^ Current diagnostic tools include smear microscopy, microbiological culture, and molecular detection by Xpert MTB/RIF (Xpert) or Xpert MTB/RIF Ultra (Ultra). These all rely on obtaining sputum or other biological samples from the site of disease. Each approach has additional limitations, such as the poor sensitivity of microscopy, the time delay for culture, the high cost of molecular tests, and false-positive Ultra results arising from detection of non-viable *Mycobacterium tuberculosis*. WHO has specified an urgent need for a rapid, simple, and low-cost triage test that prioritises sensitivity to confidently rule out tuberculosis, or to identify patients who require further investigation.^2^ A Delphi process partly informed by cost-effectiveness considerations concluded that such a test required a minimum of 90% sensitivity and 70% specificity.^2^, ^3^ As not all patients with tuberculosis produce sputum spontaneously, a nonsputum confirmatory test that prioritises specificity is also advocated.^2^

**Research in context**

**Evidence before this study**

We did a systematic review, using comprehensive terms for “tuberculosis”, “transcriptional”, “signatures”, and “blood”, with no language or date restrictions. Many studies have been done with the aim of discovering whole-blood transcriptional signatures that discriminate individuals with tuberculosis from disease-free controls or from patients with other infectious or respiratory diseases. Several candidate signatures have thus been identified, raising hope of translation into near-patient assays. However, validation of these signatures has been limited, especially in settings where they are needed most and in sick patients undergoing routine investigation for tuberculosis. Only one previous study compared the diagnostic accuracy of candidate signatures in a head-to-head analysis, but key signatures were not included, and validation relied solely on existing case-control datasets. It has therefore been unclear which candidate signature works best for the diagnosis of tuberculosis, or if any signatures meet minimum or optimum benchmarks proposed by WHO in a real-world observational cohort. Addressing these research gaps is crucial to inform whether these biomarkers should be translated into scalable test platforms or considered for adoption by national programmes.

**Added value of this study**

To our knowledge, we provide the first comprehensive and systematic head-to-head comparison of candidate transcriptional signatures for identification of active tuberculosis in a prospective diagnostic accuracy study. Moreover, we used an unbiased consecutive sampling approach, in contrast to the case-control design of previous studies. Among 181 consecutive patients presenting for investigation of presumptive pulmonary tuberculosis in South Africa, four of 27 candidate transcriptional signatures performed equivalently to each other in discriminating individuals with tuberculosis from those without, irrespective of HIV status and other baseline characteristics. These signatures met or approximated to the minimum WHO target product profile for a triage test (of 90% sensitivity, 70% specificity). However, no signature met the optimum criteria (of 95% sensitivity, 80% specificity) for a tuberculosis triage test, or the minimum criteria for a confirmatory test (65% sensitivity, 98% specificity). The best-performing signatures all improved the specificity of the Xpert MTB/RIF Ultra microbiological molecular test for *Mycobacterium tuberculosis* DNA, in which the advantages of greater sensitivity have been undermined by a higher rate of false-positive results.

**Implications of all the available evidence**

Selected blood transcriptional biomarkers show promise as triage tests for patients being investigated for pulmonary tuberculosis in high-incidence settings, exemplified by our study site. The signatures did not achieve the minimum criteria needed for a confirmatory test and should not be used by themselves for this purpose. Nonetheless, they might improve diagnostic accuracy when used in conjunction with highly sensitive molecular tests for *M tuberculosis* DNA. These data support further development of assays for blood transcriptional biomarkers to enable interventional trials of their potential clinical and health-economic effects in the diagnostic pathway for tuberculosis.

Many host blood transcriptional signatures have been proposed to differentiate patients with pulmonary tuberculosis from healthy controls or patients with other infectious or respiratory diseases,^4^ raising hopes for translation into near-patient assays. However, validation of these signatures is currently limited to evidence from case-control studies.^5^ Such studies are prone to overestimate performance because of the spectrum effect arising from differences in disease prevalence and other unmeasured covariates in selected patient subgroups, and biased inclusion of cases at extremes of the distribution of phenotypes that might not be representative of the target population.^6^ Independent validation in prospective, real-world populations is therefore crucial to assess true test performance, however, there are no comprehensive head-to-head comparisons in such settings for candidate blood transcriptional tuberculosis signatures.

WHO has endorsed use of Ultra to provide increased sensitivity compared with Xpert for PCR detection of *M tuberculosis* in sputum specimens.^7^ However, Ultra returns more false-positive results (culture-negative) than Xpert, particularly within the semi-quantitative trace output category, which detects the lowest bacillary burden of *M tuberculosis*.^8^ The large number of false-positives has been attributed to detection of DNA from non-culturable *M tuberculosis* as a result of past infection, which is more likely in high-burden settings.^9^ The decreased specificity makes diagnostic interpretation of positive Ultra results challenging, and potentially undermines the value of its greater sensitivity.^7^, ^8^, ^10^ Therefore, in addition to being applied as standalone tests, blood transcriptional biomarkers of tuberculosis could improve the specificity of Ultra by resolving results in which only traces of DNA are detected or those in patients with previous tuberculosis. We undertook a prospective observational study to compare the diagnostic accuracy of candidate transcriptional signatures identified by systematic review. Our primary objective was to benchmark the performance of the signatures against the WHO target product profile (TPP) for a tuberculosis triage test. As secondary objectives, we sought to assess the performance of these signatures against WHO TPP criteria for a blood-based confirmatory tuberculosis test, and to explore their potential use as an add-on confirmatory test to clarify interpretation of positive Ultra results.

## Methods

### Study design and participants

Our study was nested within a diagnostic accuracy study of sputum Xpert and Ultra tests for pulmonary tuberculosis.^10^ Symptomatic adults (≥18 years) self-presenting for investigation of pulmonary tuberculosis were consecutively recruited in Cape Town, South Africa, from a tuberculosis clinic within a government primary health-care centre (Scottsdene). Patients were screened and investigated according to South African guidelines.^11^ At recruitment, demographic and clinical metadata were recorded, including a modified tuberculosis symptom score (appendix 1, p1).^12^

This study was approved by the Stellenbosch University Faculty of Health Sciences Research Ethics Committee (N14/10/136). All participants provided written informed consent.

### Specimen microbiology and definitions

Blood was collected in Tempus tubes, and patients provided two sputum samples. One was decontaminated by Mycoprep (BD, Johannesburg, South Africa) before double Ziehl-Neelsen smear microscopy and Mycobacteria Growth Indicator Tube 960 liquid culture (appendix 1, p1). The second sputum sample was used for Xpert testing. The next morning, patients provided a third sputum sample for Ultra testing. Sputum samples were either obtained via spontaneous expectoration or induced by nebulising with 5% sodium chloride for 7–10 min.

In our primary analysis, patients with tuberculosis were defined as those with either a positive liquid culture or a positive Xpert result, to overcome the limitation of a single culture reference. Patients with missing blood RNA or sputum results were excluded.

### Blood RNA sequencing and data processing

Extraction and sequencing of blood mRNA was done as previously described,^13^ resulting in a median of 25 million (range 9–33 million, IQR 21–27 million) 41 bp paired-end reads per sample. Blood samples with an insufficient RNA yield were not processed for sequencing. Data are available on Array Express, accession number E-MTAB-8290. RNA sequencing and data processing were done independently of microbiological test results. RNAseq data were mapped to the reference transcriptome (Ensembl Human GRCh38 release 95) and processed as previously described,^14^ focusing on protein-coding genes. Unless otherwise specified, log_2_-transformed transcripts per million values were used for analysis. To account for an observed batch effect that could not be accounted for by any biological or known technical variables (appendix 1, p 13), we tested two batch correction techniques, using the *ComBat* and *sva* functions from the sva package in R, respectively (appendix 1, pp 1–2).^15^ Since surrogate variable analysis preserved specified outcomes of interest (tuberculosis status, HIV status, age, sex, and ethnicity) while correcting any other, unwanted variation, and because samples clustered more tightly after batch correction with surrogate variable analysis (SVA; appendix 1, p 14), we used SVA-adjusted data for the primary analyses.

### Systematic review of blood transcriptional signatures for tuberculosis

We previously did a systematic review^14^ to identify candidate concise whole-blood transcriptional signatures for incipient or active tuberculosis published before April 15, 2019, including only signatures that were discovered by comparison with asymptomatic controls. In the present study, we extended the inclusion criteria from the previous review to also capture signatures intended to distinguish active tuberculosis from other diseases (appendix 1, p 2). Additionally, following initial peer review, we included two further signatures that met the inclusion criteria but were published after the date limit of our search.^16^, ^17^ All screened articles are listed in appendix 2, with reviewed full text articles matched against inclusion criteria.

Signature scores were calculated using the original authors' methods (appendix 1, pp 2–4). Some signatures included genes whose annotations have since been withdrawn, or non-coding RNA and putative pseudogenes that were not present in our protein-coding RNAseq dataset (appendix 3). Where changes to the original model were made, or where a model had to be recreated, we validated the reconstructed model by comparing the area under the receiver operating characteristics curves (AUROCs) in the original dataset where possible (appendix 1, p 7).

### Statistical analysis

Our sample size was primarily determined by the number of participants in the parent study^10^ with paired blood RNA and sputum samples. To assess our statistical power, we used published models for estimates of sample size calculations in diagnostic tests (appendix 1, p 12).^18^, ^19^ The prevalence of tuberculosis in patients of the parent study was 30% (72/239).^10^ At this prevalence, a total sample size of more than 135 participants was required to establish whether the blood transcriptional biomarkers could achieve the minimum thresholds of the WHO TPP for a triage test (90% sensitivity and 70% specificity) with a 10% margin of error. Assuming the best-performing test achieved an AUROC of at least 0·9 (as is generally the case in the original reports of each signature), a total sample size of more than 130 participants was required for 80% power to identify a 0·1 difference in AUROCs between paired tests.

This study is reported in accordance with the Standards for Reporting of Diagnostic Accuracy Studies guidelines.^20^ p values of less than 0·05 were considered statistically significant. Cohort characteristics were compared with χ^2^ or Mann-Whitney tests. CIs for the differences between proportions were calculated using the Newcombe-Wilson method with continuity correction.^21^ The pROC package in R was used to construct receiver operating characteristic (ROC) curves to discriminate between patients with and without tuberculosis. CIs for ROC curves' sensitivities were plotted at 1% specificity intervals, using the *ci.se* function of the pROC package. We compared AUROCs for each candidate signature in a pairwise approach with the DeLong method,^22^ using the signature with highest AUROC as reference.

To test for differential diagnostic accuracy among predefined population subgroups, we stratified the cohort according to age, sex, ethnicity, HIV infection, previous tuberculosis, and indices of disease severity at presentation (symptom score, body-mass index [BMI], haemoglobin concentration, and sputum smear results). We constructed univariable subgroup-specific ROC curves and compared their AUROCs using DeLong tests. Sensitivity, specificity, and predictive values were reported at the maximum Youden index reflecting the highest test accuracy.^23^ Additionally, we assessed diagnostic accuracy when fixing sensitivity and specificity at the minimum and optimum thresholds, as defined by the WHO TPP criteria for triage and confirmatory tests of tuberculosis,^2^ using the *coords* function in the pROC package. WHO thresholds for a triage test were minimum 90% sensitivity, 70% specificity; optimum 95% sensitivity, 80% specificity. WHO thresholds for a confirmatory test were minimum 65% sensitivity, 98% specificity. CIs for proportions were calculated using the binomial Wilson method,^24^ implemented in the *binconf* function of the hmisc R package. We used the upper limit of the CIs for each signature to assess whether they might achieve the required thresholds for sensitivity and specificity. McNemar's tests were used to compare sensitivity and specificity between Ultra analysis alone and a diagnostic algorithm combining sputum Ultra analysis with transcriptional signatures (appendix 1, p 4).

We did three sensitivity analyses to confirm the robustness of our results. First, we restricted the tuberculosis case definition to patients with culture-confirmation, irrespective of Xpert results. Second, we estimated the best possible specificity of the transcriptional signatures by simulating increased sensitivity of the standard reference that might be achieved using four sputum cultures (appendix 1, p 4),^25^ as previously described.^10^ Third, *ComBat* was used as an alternative batch correction method to the surrogate variable analysis used in primary analysis. All statistical analyses were done, and data graphically visualised, in R (version 3.6.0) or GraphPad Prism (version 8.1.1).

### Role of the funding source

The funders of the study had no role in study design, data collection, data analysis, data interpretation, writing of the report, or the decision to submit for publication. The corresponding author had full access to all the data in the study and had final responsibility for the decision to submit for publication.

## Results

**Figure 1:**
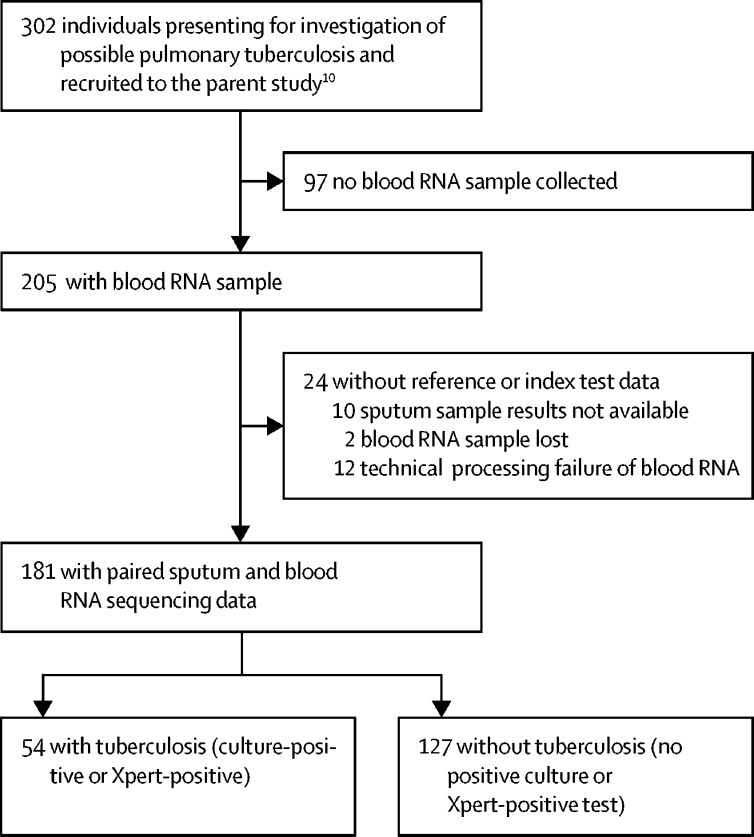
Study flowchart

Between Feb 12, 2016, and July 18, 2017, we obtained blood RNA samples from 205 consecutive patients.^10^ Paired sputum and RNA sequencing data were available in 181 participants included in our analysis (figure 1). Their baseline characteristics are given in table 1; characteristics of participants who were excluded from the analysis are in appendix 1 (p 8). 54 (30%) of 181 patients had pulmonary tuberculosis, confirmed by sputum culture or Xpert, and included all the individuals who received tuberculosis treatment at enrolment, further increasing our confidence in the sensitivity of our standard reference for the diagnosis of tuberculosis (appendix 1, p 15). 44 (24%) of 181 patients were HIV-infected.

**Table 1:** Baseline characteristics of study cohort

		**All (n=181)**	**No tuberculosis (n=127)**	**Positive for pulmonary tuberculosis (n=54)**
Age, years		35 (27–48)	36 (28–49)	34 (24–43)
Sex				
	Male	94 (52%)	66 (52%)	28 (52%)
	Female	87 (48%)	61 (48%)	26 (48%)
Ethnicity				
	Black	28 (15%)	14 (11%)	14 (26%)
	Mixed ancestry	153 (85%)	113 (89%)	40 (74%)
HIV status				
	Unknown*	1 (1%)	1 (1%)	0
	Uninfected	136 (75%)	99 (78%)	37 (69%)
	Infected	44 (24%)	27 (21%)	17 (31%)
Antiretroviral therapy†				
	No	24 (55%)	14 (52%)	10 (59%)
	Yes	15 (34%)	12 (44%)	3 (18%)
	Unknown*	5 (11%)	1 (4%)	4 (24%)
CD4 count†, cells per μL		334 (192–606)	354 (207–707)	326 (128–484)
Haemoglobin concentration, g/dL		13·7 (12·4–14·8)	14·2 (13·2–15·4)	12·6 (11·3–13·6)
Leucocyte count, × 10^9^ cells per L		8 (6·1–10·2)	7·6 (6–9·8)	9·1 (6·8–11)
BMI, kg/m^2^		19·9 (17·8–22·5)	20·5 (18·4–23·2)	19·1 (16·8–21·5)
Tuberculosis symptom score		2 (2–3)	2 (1–3)	3 (2–5)
Previous tuberculosis				
	No	115 (64%)	81 (64%)	34 (63%)
	Yes	66 (36%)	46 (36%)	20 (37%)
Liquid culture				
	Positive	53 (29%)	NA	53 (98%)
	Negative	128 (71%)	128 (100%)	1 (2%)
Sputum smear				
	Positive	15 (8%)	1 (1%)	14 (26%)
	Negative	157 (87%)	120 (94%)	37 (69%)
	Not done	9 (5%)	6 (5%)	3 (6%)
Xpert				
	Positive	44 (24%)	NA	44 (81%)
	Negative	134 (74%)	124 (98%)	10 (19%)
	No result	2 (2%)	2 (2%)	NA
	Not done	1 (1%)	1 (1%)	NA
Ultra				
	Positive‡	51 (28%)	10 (8%)	41 (76%)
	Negative	103 (57%)	94 (74%)	9 (17%)
	No result	10 (6%)	8 (6%)	2 (4%)
	Not done	17 (9%)	15 (12%)	2 (4%)

Data are n (%) or median (IQR). Individuals positive for pulmonary tuberculosis were defined as those with either a positive liquid culture or a positive Xpert MTB/RIF result, or both. Individuals with missing data: CD4 cell counts (n=1), haemoglobin concentration (n=3), leucocytes (n=3), BMI (n=1), symptom score (n=3). BMI=body-mass index. NA=not applicable.

* Category excluded for χ^2^ statistical test.

† Antiretroviral therapy and CD4 cell counts for HIV-infected patients only.

‡ Positive Ultra results include tests where traces of *Mycobacterium tuberculosis* were detected.

Compared with individuals without tuberculosis, a greater proportion of patients with tuberculosis were black (difference of proportions 0·24 [95% CI 0·04–0·44]). Patients with tuberculosis also had higher symptom scores (difference of means 1·1, [0·6–1·5]), lower haemoglobin concentrations (−1·7 [–2·3 to −1·1]), lower BMI (−1·9 [–3·3 to −0·4]), and increased leucocytes (1·3 [0·2–2·4]). None of these clinical parameters independently discriminated between patients with and without tuberculosis with sufficient diagnostic accuracy for a tuberculosis triage test as defined by WHO TPP (appendix 1, p 16).^2^

27 signatures from 18 of 645 articles identified by our systematic review and expert consultation met the inclusion criteria (appendix 1, p 17; appendix 2; table 2). 14 of these 27 signatures were derived from study populations that included South African participants. Ten signatures were discovered in datasets that included HIV-infected participants. Five signatures were intended for diagnosis of incipient tuberculosis; 22 signatures were discovered with their intended application for diagnosis of active tuberculosis disease. Of these 22 signatures, eight aimed to distinguish tuberculosis from asymptomatic controls (including people who were healthy or with latent tuberculosis infection), five targeted discrimination of tuberculosis from other diseases, and nine aimed to distinguish tuberculosis from a mixed population of patients with other diseases and healthy controls. 24 of the 27 signatures were discovered through a genome-wide approach. Ten signatures required reconstruction of random forest or support vector machine models. We assessed whether each of the models that required reconstruction or had been otherwise altered, achieved the AUROC reported by the authors in the original dataset (appendix 1, p 7). We could not recapitulate the original AUROC for two signatures: Anderson39.OD,^26^ which had been reduced from 51 transcripts originally to the 39 protein-coding transcripts that were available in our RNAseq dataset, and Duffy10,^16^ for which our attempt to reconstruct the original model did not achieve the same AUROC as that reported in their validation data. In this case, we used a binary support vector machine model, which did reproduce a similar AUROC in their validation cohort. In addition, this assessment was not possible for two other signatures (Huang11 and Kaforou45) for which the AUROCs were not provided in the original reports.^30^, ^31^

**Table 2:** Description of candidate blood transcriptional signatures for tuberculosis

	**Original gene number**	**Model**	**Intended application**	**Discovery datasets**						
				Population	HIV status	Setting	Approach	Tuberculosis cases	Controls	Total
Anderson39.LTBI^26^	42	Disease risk score	Tuberculosis *vs* LTBI	Children	Positive or negative	South Africa, Malawi	Elastic net using genome-wide data	87	43	130
Anderson39.OD^26^	51	Disease risk score	Tuberculosis *vs* OD	Children	Positive or negative	South Africa, Malawi	Elastic net using genome-wide data	87	134	221
BATF2^27^	1	NA	Tuberculosis *vs* HC (acute *vs* convalescent)	Adults	Negative	UK	SVM using genome-wide data	46	31	77
Duffy10^16^	10	SVM (linear kernel)	Tuberculosis *vs* LTBI and OD	Adults	Positive or negative	South Africa	Multinomial random forest using genome-wide data	93	207	300
Gjoen8^28^	7	LASSO regression	Tuberculosis *vs* HC and OD	Children	Negative	India	LASSO using 198 pre-selected genes	47	36	83
Gliddon3^29^	3	(FCGR1A + C1QB) − (ZNF296)	Tuberculosis *vs* LTBI	Adults	Positive or negative	South Africa, Malawi	FS-PLS using genome-wide data	NS	NS	285
Gliddon4^29^	4	(GBP6 + PRDM1) − (TMCC1 + ARG1)	Tuberculosis *vs* OD	Adults	Positive or negative	South Africa, Malawi	FS-PLS using genome-wide data	NS	NS	293
Huang11^30^	13	SVM (linear kernel)	Tuberculosis *vs* HC and OD	Adults	Negative	UK	Common genes from elastic net, L1/2 and LASSO models, using genome-wide data	16	79	95
Kaforou25^31^	27	Disease risk score	Tuberculosis *vs* LTBI	Adults	Positive or negative	South Africa, Malawi	Elastic net using genome-wide data	NS	NS	285
Kaforou39^31^	44	Disease risk score	Tuberculosis *vs* OD	Adults	Positive or negative	South Africa, Malawi	Elastic net using genome-wide data	NS	NS	293
Kaforou45^31^	53	Disease risk score	Tuberculosis *vs* LTBI and OD	Adults	Positive or negative	South Africa, Malawi	Elastic net using genome-wide data	NS	NS	NS
Maertzdorf4^32^	4	Random forest	Tuberculosis *vs* HC	Adults	Negative	India	Random forest using 360 selected target genes	113	76	189
NPC2^33^	1	NA	Tuberculosis *vs* HC and LTBI	Adults	NS	Brazil	Differential expression using genome-wide data	6	28	34
Penn-Nicholson6^17^	6	Difference of means	Incipient tuberculosis *vs* HC	Adolescents	Negative	South Africa	SVM-based gene pair models using genome-wide data	46	107	153
Qian17^34^	17	Sum of standardised expression	Tuberculosis *vs* HC and OD	Adults	Negative	UK	Differential expression of Nrf2-mediated genes	16	69	85
Rajan5^35^	5	Unsigned sums	Tuberculosis *vs* HC (screening among PLHIV)	Adults	Positive	Uganda	Differential expression using genome-wide data	NS	NS	80 (1:2 cases:controls)
Roe3^13^	3	SVM (linear kernel)	Incipient tuberculosis *vs* HC	Adults	Negative	UK	Stability selection using genome-wide data	46	31	77
Roe4^27^	4	SVM (linear kernel)	Tuberculosis *vs* OD	Adults	Negative	UK	SVM using genome-wide data	23	35	58
Roe5^27^	5	SVM (linear kernel)	Tuberculosis *vs* HC and OD	Adults	Negative	UK	SVM using genome-wide data	23	50	73
Singhania20^36^	20	Modified disease risk score	Tuberculosis *vs* HC and OD	Adults	Negative	UK, South Africa	Random forest using modular approach	NS	NS	NS
Suliman2^37^	2	ANKRD22 −OSBPL10	Incipient tuberculosis *vs* HC	Adults	Negative	The Gambia, South Africa	Pair ratios algorithm using genome-wide data	79	328	407
Suliman4^37^	4	(GAS6 + SEPT4) – (CD1C + BLK)	Incipient tuberculosis *vs* HC	Adults	Negative	The Gambia, South Africa, Ethiopia	Pair ratios algorithm using genome-wide data	45	141	186
Sweeney3^38^	3	(GBP5 + DUSP3)/2 −KLF2	Tuberculosis *vs* LTBI and OD	Adults	Positive or negative	Meta-analysis of South Africa, Malawi, UK, France, USA	Significance thresholding and forward search in genome-wide data	296	727	1023
Walter46^39^	51	SVM (linear kernel)	Tuberculosis *vs* LTBI	Adults	Negative	USA	SVM using genome-wide data	24	24	48
Walter32^39^	47	SVM (linear kernel)	Tuberculosis *vs* OD	Adults	Negative	USA	SVM using genome-wide data	24	24	48
Walter101^39^	119	SVM (linear kernel)	Tuberculosis *vs* LTBI and OD	Adults	Negative	USA	SVM using genome-wide data	24	48	72
Zak16^40^	16	SVM (linear kernel)	Incipient tuberculosis *vs* HC	Adolescents	Negative	South Africa	SVM-based gene pair models using genome-wide data	37	77	114

Signatures were identified by systematic literature review and included for analysis. Signature names represent the first author's name of the corresponding publication, suffixed with the number of constituent genes that are present in the current RNAseq dataset. Both Anderson signatures resulted in the same number of final genes; these signatures were therefore additionally appended with the comparator control group. Details on how models were recreated are in appendix 1 (pp 2-4). LTBI=latent tuberculosis infection. OD=other diseases. NA=not applicable. HC=healthy controls. SVM=support vector machine. LASSO=least absolute shrinkage and selection operator. FS-PLS=forward selection-partial least squares. NS=not specified. Nrf2=nuclear factor, erythroid 2-like 2. PLHIV=people living with HIV.

We ranked the 27 candidate transcriptional signatures by their AUROC for discriminating tuberculosis and no tuberculosis in all 181 patients. The signature with the highest diagnostic accuracy was Sweeney3 (AUROC 90·6% [95% CI 85·6–95·6]), which was derived from an analysis of multiple previously published studies of patients with pulmonary tuberculosis compared with controls comprising both healthy individuals and patients with non-tuberculosis diseases.^41^ Pairwise comparison of the remaining 26 signatures against Sweeney3 showed that three other signatures had equivalent AUROCs. These were Kaforou25 (86·9% [80·9–92·9]), Roe3 (86·9% [80·3–93·5]), and BATF2 (86·8% [80·6–93·1]; appendix 1, p 9) all derived from individual case-control studies comparing patients with tuberculosis with healthy controls.^13^, ^27^, ^31^ The remaining 23 signatures had inferior AUROCs.

**Figure 2:**
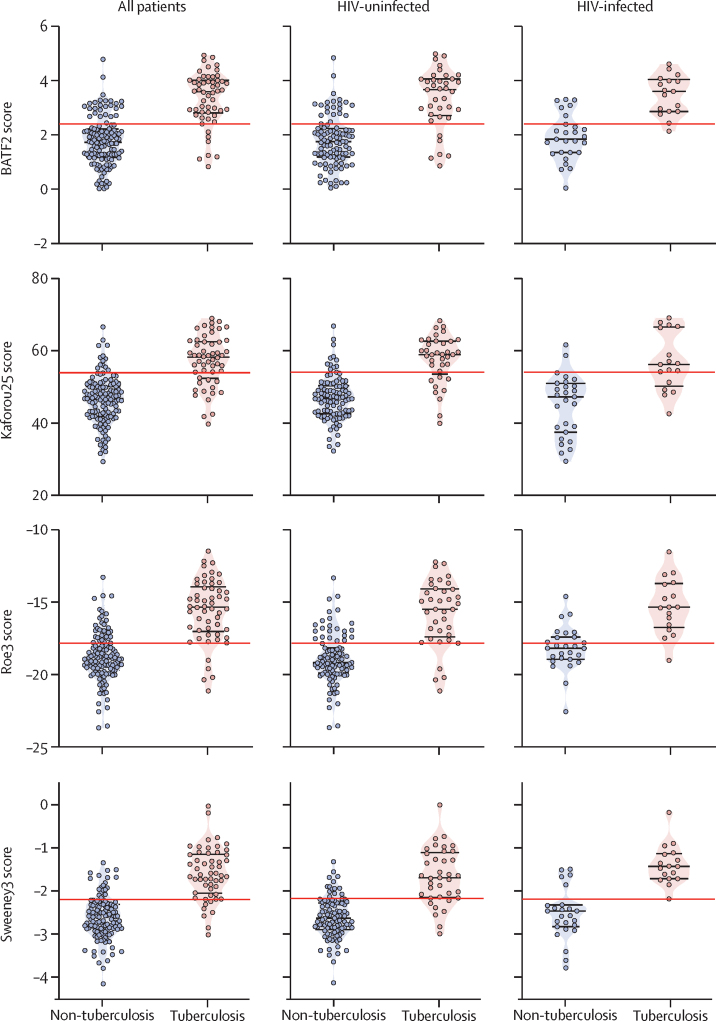
Tuberculosis scores of the four transcriptional signatures with the highest diagnostic accuracy overall and stratified by HIV status Red lines represent the score threshold of the maximal Youden index, identified from analysis of all patients. The score difference between individuals with and without tuberculosis was significant for all four signatures in both the total cohort and in HIV-stratified cohort subsets (Mann-Whitney test p<0·0001).

**Figure 3:**
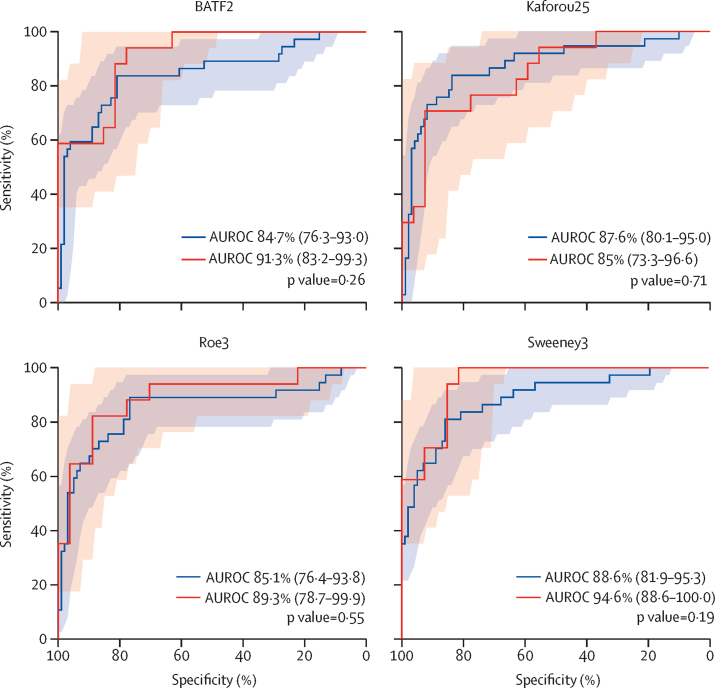
ROC curves for the four transcriptional signatures with the highest diagnostic accuracy in HIV-infected versus HIV-uninfected patients Shaded areas represent the 95% CI of the ROC curve sensitivities, plotted at 1% specificity intervals (red shading represents HIV-infected patients and blue shading represents HIV-uninfected patients). AUROC values are reported with 95% CIs in brackets. p values are derived from pairwise comparison of ROC curves, using DeLong tests. AUROC values and CIs are also in appendix 1 (p 10). ROC=receiver operating characteristic. AUROC=area under the ROC curve.

Test scores of the four signatures with the highest diagnostic accuracy, among all patients and stratified by HIV status, are shown in figure 2. In exploratory subgroup analyses, diagnostic accuracy of these four signatures was not affected by HIV infection (figure 3) or any other patient baseline characteristics, including age, sex, and previous tuberculosis disease (appendix 1, p 10). AUROCs tended to be numerically lower among black patients (compared with those of mixed ancestry), and numerically lower in patients with higher BMI and with tuberculosis symptom scores of less than 3, which might indicate less severe disease. None of these differences was significant for all four signatures. Additionally, there was no systematic effect of sputum smear status or haemoglobin concentration, as other markers of disease severity, on signature performance (appendix 1, p 10). Similarly, signature scores did not correlate with duration of cough, time to culture positivity, or minimum Xpert cycle threshold, as surrogate measures of bacterial load (appendix 1, p 18).

**Figure 4:**
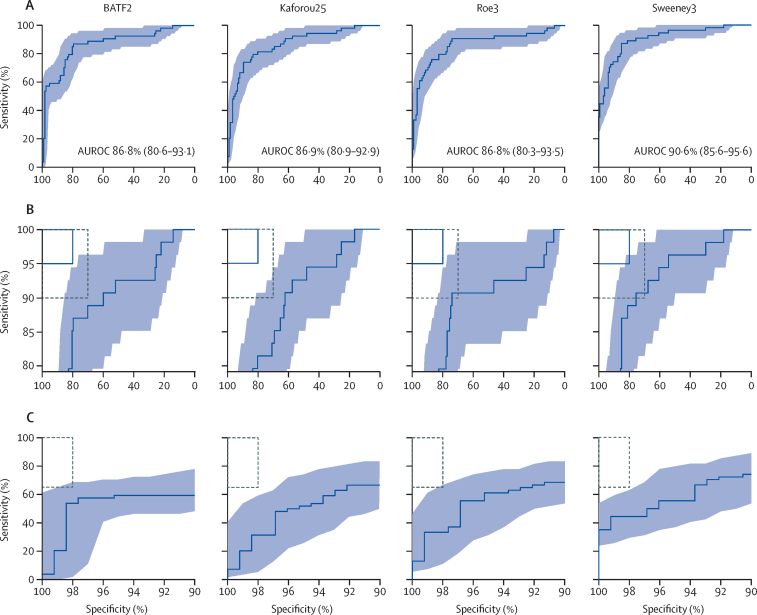
ROC curves of the four transcriptional signatures with the highest diagnostic accuracy benchmarked against WHO target product profile criteria (A) Blue shaded areas represent the 95% CIs of the ROC curve sensitivities, plotted at 1% specificity intervals. AUROC values are reported with 95% CIs in brackets. (B) ROC curves are replicated with restricted y axes, and benchmarked against target criteria for a tuberculosis triage test. Minimum criteria (90% sensitivity, 70% specificity) are indicated by the dashed black boxes, optimum criteria (95% sensitivity, 80% specificity) are indicated by the blue boxes. Light blue shaded areas represent the 95% CIs. (C) ROC curves are replicated with restricted x axes and benchmarked against minimum criteria for a confirmatory test (dashed black box: 65% sensitivity, 98% specificity). Light blue shaded areas represent the 95% CIs. ROC=receiver operating characteristic. AUROC=area under the ROC curve.

Table 3 shows the sensitivity and specificity of the BATF2, Kaforou25, Roe3, and Sweeney3 signatures at the maximum Youden index of each in all 181 patients. When ROC curves of these signatures were benchmarked against the WHO TPP criteria for a tuberculosis triage test, point estimates or 95% CIs of all four signatures reached the minimum cutoffs of 90% sensitivity and 70% specificity (figure 4). Similarly, when fixing either sensitivity at 90% or specificity at 70% to enforce minimum WHO TPP diagnostic criteria, all four signatures met or approximated to the required performance thresholds (table 3). However, the optimum target criteria of 95% sensitivity and 80% specificity were beyond the 95% CI of all four signatures, either at the maximum Youden index or when fixing sensitivity or specificity at the required thresholds (figure 4, table 3).

**Table 3:** Performance metrics of the four candidate blood transcriptional signatures with the highest diagnostic accuracy

	**Sensitivity**	**Specificity**	**PPV**	**NPV**
**At maximum Youden index**				
BATF2	87·0% (75·6–93·6)	79·5% (71·7–85·6)	64·4% (52·9–74·4)	93·5% (87·2–96·8)
Kaforou25	74·1% (61·1–83·9)	89·8% (83·3–93·9)	75·5% (62·4–85·1)	89·1% (82·5–93·4)
Roe3	90·7% (80·1–96·0)	74·0% (65·8–80·9)	59·8% (48·9–69·7)	94·9% (88·7–97·8)
Sweeney3	87·0% (75·6–93·6)	85·0% (77·8–90·2)	71·2% (59·4–80·7)	93·9% (88·0–97·0)
**At minimum sensitivity for a triage test**				
BATF2	90%	59·8% (51·1–68·0)	48·8% (39·2–58·5)	93·4% (85·8–97·0)
Kaforou25	..	62·2% (53·5–70·2)	50·3% (40·5–60·1)	93·6% (86·3–97·2)
Roe3	..	74·0% (65·8–80·9)	59·6% (48·7–69·5)	94·6% (88·2–97·6)
Sweeney3	..	75·6% (67·4–82·2)	61·1% (50·1–71·0)	94·7% (88·5–97·6)
**At minimum specificity for a triage test**				
BATF2	88·9% (77·8–94·8)	70%	55·7% (45·2–65·8)	93·7% (86·9–97·1)
Kaforou25	83·3% (71·3–91·0)	..	54·2% (43·5–64·4)	90·8% (83·4–95·1)
Roe3	90·7% (80·1–96·0)	..	56·3% (45·8–66·2)	94·7% (88·1–97·7)
Sweeney3	90·7% (80·1–96·0)	..	56·3% (45·8–66·2)	94·7% (88·1–97·7)
**At optimum sensitivity for a triage test**				
BATF2	95%	25·2% (18·5–33·4)	35·1% (27·8–43·1)	92·2% (78·6–97·5)
Kaforou25	..	28·3% (21·2–36·7)	36·1% (28·6–44·2)	93·0% (80·6–97·7)
Roe3	..	13·4% (8·5–20·4)	31·8% (25·1–39·3)	86·3% (65·3–95·5)
Sweeney3	..	54·3% (45·7–62·7)	46·9% (37·8–56·2)	96·2% (89·0–98·8)
**At optimum specificity for a triage test**				
BATF2	85·2% (73·4–92·3)	80%	64·4% (52·8–74·5)	92·7% (86·3–96·3)
Kaforou25	81·5% (69·2–89·6)	..	63·4% (51·6–73·8)	91·0% (84·3–95·1)
Roe3	79·6% (67·1–88·2)	..	62·9% (51·0–73·3)	90·2% (83·4–94·5)
Sweeney3	88·9% (77·8–94·8)	..	65·4% (54·0–75·3)	94·4% (88·4–97·4)
**At minimum sensitivity for a confirmatory test**				
BATF2	65%	85·8% (78·7–90·8)	66·1% (52·7–77·4)	85·2% (78·0–90·3)
Kaforou25	..	92·1% (86·1–95·7)	77·8% (63·8–87·5)	86·1% (79·3–90·9)
Roe3	..	92·1% (86·1–95·7)	77·8% (63·8–87·5)	86·1% (79·3–90·9)
Sweeney3	..	93·7% (88·1–96·8)	81·4% (67·4–90·3)	86·3% (79·6–91·1)
**At minimum specificity for a confirmatory test**				
BATF2	53·7% (40·6–66·3)	98%	91·9% (77·3–97·4)	83·3% (76·5–88·4)
Kaforou25	31·5% (20·7–44·7)	..	87·0% (66·0–95·8)	77·1% (70·0–82·9)
Roe3	33·3% (22·2–46·6)	..	87·6% (67·4–96·1)	77·6% (70·5–83·3)
Sweeney3	44·4% (32–57·6)	..	90·4% (73·7–97·0)	80·6% (73·6–86·0)

Data are % (95% CI). WHO defines target product profile criteria for a tuberculosis triage test as minimum 90% sensitivity and 70% specificity, optimum 95% sensitivity and 80% specificity, and for a confirmatory test as minimum 98% specificity and 65% sensitivity. PPV=positive predictive value. NPV=negative predictive value.

As a secondary objective, we assessed signature performance as a blood-based confirmatory tuberculosis test, using WHO TPP criteria as a reference (figure 4).^2^ At the maximum Youden index, all four signatures with the highest diagnostic accuracy failed to reach the required 98% specificity (table 3). Similarly, when setting the test thresholds to enforce either 98% specificity or 65% sensitivity, these four signatures were substantially short of the minimum performance requirements (table 3).

**Figure 5:**
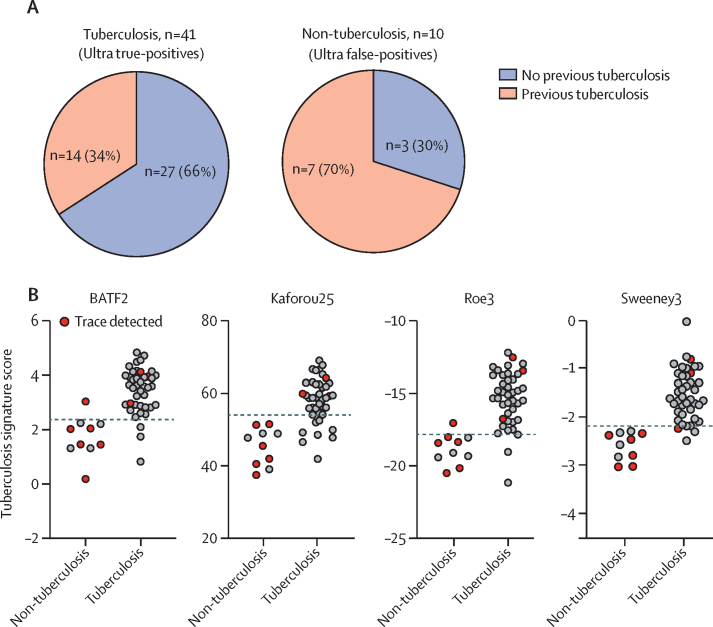
Tuberculosis scores of the four transcriptional signatures with the highest diagnostic accuracy in patients with Ultra-positive results Patients with Ultra-positive results were grouped as true-positive tuberculosis (culture-positive or Xpert-positive) and false-positive non-tuberculosis (culture-negative or Xpert-negative). (A) Pie charts representing the proportion of Ultra-positive patients with tuberculosis and individuals without tuberculosis with a history of previous tuberculosis disease. (B) Scores of the four transcriptional signatures with the highest diagnostic accuracy in patients with Ultra-positive results. Red dots indicate patients for whom only traces of *Mycobacterium tuberculosis* were detected by Ultra analysis. Dashed lines represent the score thresholds of the maximum Youden index, identified from analysis of all patients. The score difference between patients with and without tuberculosis was significant for all four signatures (Mann-Whitney test p<0·0001).

In view of emerging concerns that the higher sensitivity of the Ultra test might be compromised by false-positive results,^7^, ^8^, ^10^ we also assessed the potential use of blood signatures as an add-on confirmatory test for Ultra-positive patients. Of 51 patients with Ultra-positive results in our cohort, ten (20%) were designated as false-positive by comparison with our standard reference (ie, these individuals were culture-negative and Xpert-negative at enrolment). Six (60%) of the ten Ultra false-positive patients had trace-positive results. Previous tuberculosis disease was more common in patients with Ultra false-positive results compared with patients with Ultra true-positive results (seven [70%] of ten *vs* 14 [34%] of 41; χ^2^ test p=0·039; figure 5).

Nine of the ten Ultra false-positive patients scored consistently below the Youden index threshold of all four transcriptional signatures with the highest diagnostic accuracy, correctly classifying them as non-tuberculosis (figure 5). This also included five of the six Ultra trace false-positives. However, two to eight (5–20%) of the 41 true-positive Ultra patients were incorrectly classified as non-tuberculosis at the Youden index threshold of each signature, consistent with the imperfect sensitivity of the transcriptional signatures. A diagnostic algorithm that used the blood transcriptional signature results to re-classify all Ultra-positive patients, or only those with trace results, or those with previous tuberculosis, led to improved specificity compared with Ultra analysis alone, with small associated reductions in sensitivity (table 4). Of note, follow-up of cases that were Ultra-positive but culture-negative in the parent study revealed that three of the ten cases that we designated as Ultra false-positives were diagnosed with tuberculosis at intervals of 295, 432, and 777 days.^10^

**Table 4:** Sensitivity and specificity of a diagnostic algorithm combining the sputum Ultra test with blood transcriptional signature analysis

	**Ultra test alone**	**Addition of signature to:**		
		All Ultra-positive individuals	Ultra trace-positive individuals	Ultra-positive individuals with previous tuberculosis
**Sensitivity**				
BATF2	82% (69–90)	76% (63–86)	82% (69–90)	80% (67–89)
Kaforou25	..	66% (52–78); p=0·013	82% (69–90)	74% (60–84)
Roe3	..	78% (65–87)	82% (69–90)	80% (67–89)
Sweeney3	..	76% (63–86)	80% (67–89)	78% (65–87)
**Specificity**				
BATF2	90% (83–95)	99% (95–100); p=0·0077	95% (89–98)	96% (91–98); p=0·041
Kaforou25	..	100% (96–100); p=0·0044	96% (91–98); p=0·041	97% (92–99); p=0·023
Roe3	..	99% (95–100); p=0·0077	95% (89–98)	96% (91–98); p=0·041
Sweeney3	..	100% (96–100); p=0·0044	96% (91–98); p=0·041	97% (92–99); p=0·023

Data are % (95% CI). Only significant p values are shown. Sensitivity and specificity were calculated for 154 patients with Ultra results, with or without reclassification of selected Ultra-positive tests by transcriptional signatures. p value of comparison with Ultra alone using McNemar's test.

Restricting the tuberculosis case definition to culture-proven patients led to re-assignment of only one culture-negative, Xpert-positive patient as without tuberculosis. Data reanalysis confirmed the finding that the four signatures performed equivalently, independent of HIV status, while meeting or approximating the minimum criteria for a tuberculosis triage, but not confirmatory, test (appendix 1, pp 19–20). The possibility that some patients might have been diagnosed with tuberculosis after enrolment to our study and the absence of multiple sputum cultures might have led to an underestimation of the specificity of the transcriptional signatures. To overcome this limitation, we sought to estimate the best possible specificity that the signatures could achieve if the sensitivity of the standard reference was increased by additional sputum cultures. We reclassified signature false-positive cases (at the Youden index threshold) to true-positive cases by the ratio of the sensitivity expected from four sputum cultures to that of a single culture.^25^ Even in this analysis, the four signatures with the highest diag-nostic accuracy failed to achieve optimum criteria for a triage test, and minimum criteria for a confirmatory test (appendix 1, p 11). Finally, we repeated our analysis after batch correction with *ComBat* instead of surrogate variable analysis. Again, our main findings were unchanged, confirming the robustness of our results (appendix 1, pp 21–22).

## Discussion

To our knowledge, this is the first comprehensive head-to-head analysis of candidate blood transcriptional biomarkers of tuberculosis in a prospective validation cohort with a high burden of tuberculosis and HIV. Four signatures (comprising 1–25 genes) had equivalent diagnostic accuracy for differentiating patients with and without tuberculosis, irrespective of HIV status. These signatures met or approximated to the minimum WHO TPP criteria of 90% sensitivity and 70% specificity for a triage test to rule out tuberculosis, but failed to reach the optimum criteria (95% sensitivity and 80% specificity), and at a test threshold that offers the maximum diagnostic accuracy, they missed 9–26% of tuberculosis cases (ie, five to 14 of 54 patients with tuberculosis).

To date, no transcriptional signature has been translated into a point-of-care test, which would require the adaptation and validation of these tests as PCR-based assays. Such studies are underway;^17^, ^29^ however, the cost is likely to exceed the target threshold of $2 per sample.^2^ Taken together with the suboptimal clinical performance observed in our study, the question is raised of whether host transcriptional biomarkers represent a realistic and achievable triage strategy for the resource-limited settings where they are most needed. Of note, the diagnostic accuracy of the best transcriptional biomarkers in the current analysis was similar to that of point-of-care C-reactive protein (CRP) alone for active case-finding among HIV-infected individuals.^42^ Since CRP testing is likely to be substantially cheaper, prospective assessments of the superiority of transcriptional biomarkers above this benchmark are required if they are to be pursued for this application. We also tested whether transcriptional biomarkers could be used as blood-based confirmatory tests for tuberculosis, for which the WHO-specified maximum target price is higher. However, the transcriptional signatures with the highest diagnostic accuracy in our study had insufficient specificity, making them non-viable for confirmatory tuberculosis diagnostics. A principal advantage of these signatures is the easy accessibility of blood sampling. However, alternative microbiological tests for tuberculosis using non-sputum samples are being developed,^43^, ^44^ which might offer greater promise among patient subgroups where obtaining sputum is difficult.

In the current cohort, ten patients had false-positive Ultra results, including six with false-positive Ultra trace results. This finding permitted exploration of alternative clinical applications of host transcriptional signatures. The four signatures with the highest diagnostic accuracy in our study showed promise in correctly classifying Ultra false-positive patients, including those with trace results. Our preliminary results suggest that a diagnostic algorithm combining Ultra sputum analysis with blood transcriptional biomarkers improves Ultra specificity. Large-scale prospective validation studies are required to further assess this potential application, particularly among individuals suspected to be false-positives, such as those with trace results or a history of tuberculosis disease. Of note, Ultra false-positive results have been attributed to non-viable mycobacterial remnants,^7^, ^8^, ^10^ but the fact that three individuals with Ultra false-positive results were diagnosed with tuberculosis after 295–777 days' follow-up raises the possibility that some false-positive results might represent detection of very early paucibacillary or latent infection, undetected by Xpert or culture. In a high-burden setting, we cannot exclude the possibility that these cases were due to acquisition of infection after enrolment. Therefore, whether Ultra-positive results in the absence of prevalent disease predict future incident disease, can only be addressed by randomised trials to test whether tuberculosis treatment in this group will reduce incident disease.

Among the best-performing signatures, BATF2, Kaforou25, and Roe3 were originally discovered by comparing patients with active tuberculosis with asymptomatic individuals.^13^, ^27^, ^31^ Nonetheless, their performance in this observational cohort of almost exclusively symptomatic patients suggests that these signatures can discriminate between tuberculosis and the casemix of other symptomatic illness in this context. Assessing the extent to which these findings are generalisable will require similar observational studies in settings that might have a different casemix. Additionally, whether existing signatures have reached the maximum possible diagnostic accuracy using blood transcriptomics, or whether novel signatures, derived on even larger discovery datasets, might lead to further improvements in diagnostic accuracy, remains to be seen. Likewise, whether integration of clinical metadata with biomarkers will generate models with greater diagnostic accuracy also needs to be tested using independent training and validation cohorts.

Within the limitations of the statistical power in our cohort, signature performance was independent of age, sex, HIV coinfection, or previous tuberculosis disease, and preserved in subgroup analyses of patients stratified by sputum smear status or haemoglobin concentrations as surrogate measures of disease severity. The point estimates for test performance among black patients, and patients with higher BMI and lower tuberculosis symptom scores were lower, but our study had insufficient power to assess the significance of these observations for all four signatures with the highest diagnostic accuracy.

An important strength of our study was the clinically relevant, real-life population of patients who were evaluated for tuberculosis in a high-burden setting, with both HIV-infected and HIV-uninfected individuals, and patients with varying severity of tuberculosis disease. We induced sputum, ensuring that we did not include only patients who could expectorate, for whom there is less need for non-sputum tests. Importantly, the non-tuberculosis group was not pre-selected to be homo-genous, thus likely encompassing a casemix of people with latent tuberculosis infection and other diseases. Second, we used a robust standard of culture or Xpert positivity as a diagnostic reference for our primary analysis, and confirmed that the most optimistic estimates of additional cultures in the standard reference would not significantly improve signature performance. Third, we did a systematic review to identify 27 candidate transcriptional signatures for tuberculosis to undertake a comprehensive head-to-head analysis. Finally, our dataset was exclusively used for validation rather than discovery, making it a truly independent diagnostic accuracy study.

A limitation of our study was the observed batch effect in RNA sequencing data, which appeared to result from a mixture of technical batch factors. We addressed this effect with two different data adjustment approaches, and found in both analyses that the same four signatures performed equivalently, irrespective of HIV status, and met or approximated the minimum criteria for a tuberculosis triage but not a confirmatory test. A second limitation of our study was that our cohort was restricted to adults with possible pulmonary tuberculosis. Similar independent validation studies are needed for children and patients with extrapulmonary tuberculosis. Since inclusion criteria for our systematic review were not limited by age or site of disease, the 27 candidate signatures identified could be tested in such a study. Third, no alternative diagnoses were available for patients without tuberculosis; thus, we were not able to establish whether false-positive results were related to particular non-tuberculosis diseases. Finally, this study was limited to transcriptional biomarkers. Prospective head-to-head studies comparing performance of transcriptional signatures with other candidate triage test biomarkers, such as point-of-care CRP^42^ and automated chest radiograph interpretation tools,^45^ or with strategies that integrate biomarkers with clinical metadata, are needed.

In conclusion, we showed that four blood transcriptional signatures have equivalent diagnostic accuracy for active tuberculosis, independent of HIV status. These biomarkers achieved the WHO minimum diagnostic accuracy parameters required for a tuberculosis triage test but failed to meet the criteria for a confirmatory test in the present setting. Notwithstanding the challenge of achieving the desired target price for such tests, further validation studies are needed to assess their application in different settings alongside head-to-head comparisons with other candidate triage biomarkers, with a view to interventional trials to assess their clinical and health economic effects.
