# Supplementary material for: Blood transcriptional biomarkers for active pulmonary tuberculosis in a high-burden setting: a prospective, observational, diagnostic accuracy study
Source: Lancet Respir Med. 2020 Apr;8(4):407–19. doi: 10.1016/S2213-2600(19)30469-2 (PMC7113842; doi:10.1016/S2213-2600(19)30469-2)
Supplement: Supplementary appendix 1 [file mmc1.pdf]

THE LANCET
Respiratory Medicine

Supplementary appendix 1

This appendix formed part of the original submission and has been peer reviewed. We post it as supplied by the authors.

Supplement to: Turner CT, Gupta RK, Tsaliki E, et al. Blood transcriptional biomarkers for active pulmonary tuberculosis in a high-burden setting: a prospective, observational, diagnostic accuracy study. *Lancet Respir Med* 2020; published online March 13. https://doi.org/10.1016/S2213-2600(19)30469-2.

Systematic validation of blood transcriptional biomarkers for active pulmonary tuberculosis in a high-burden setting: a prospective diagnostic accuracy study

Supplementary Appendix 1

Supplementary Methods **1**

Study eligibility criteria 1

TB symptom score 1

Sputum culture 1

Batch correction of RNA sequencing data 1

Eligibility criteria for candidate signatures identified through systematic review 2

Calculation of signature scores 2

Diagnostic algorithm combining sputum Ultra analysis with blood transcriptional signatures 4

Best-case four-culture simulation 4

References **5**

Supplementary Tables **7**

Table S1. Signature validation against the authors' original descriptions by comparing area under the receiver operating characteristic curve in common datasets. 7

Table S2. Baseline characteristics of patients included in and excluded from the study subset. 8

Table S3. Area under the receiver operating characteristic curve of candidate transcriptional signatures for tuberculosis. 9

Table S4. Area under the receiver operating characteristic curve of the four best-performing blood transcriptional signatures in subgroup analyses. 10

Table S5. Diagnostic performance metrics at the maximum Youden index in a best-case four-culture simulation analysis. 11

Supplementary Figures **12**

Figure S1. Estimates of sample size and power. 12

Figure S2. Principal component analysis of gene expression data shows separation of samples along the first principal component which is not explained by biological or known technical variables. 13

Figure S3. Principal component analysis of unadjusted gene expression data, and after batch correction by surrogate variable analysis or ComBat. 14

Figure S4. Venn diagram visualizing concordance between culture-positivity, Xpert-positivity and initiation of tuberculosis treatment. 15

Figure S5. Receiver operating characteristic curves of clinical characteristics, benchmarked against World Health Organization Target Product Profile criteria for a tuberculosis triage test. 16

Figure S6. Flowchart showing systematic review process to identify concise whole blood transcriptional signatures for active or incipient tuberculosis. 17

Figure S7. Tuberculosis signature scores do not correlate with bacterial load or symptom duration. ... 18

Figure S8. Sensitivity analysis with tuberculosis case definition restricted to culture-proven patients. 19

Figure S9. Sensitivity analysis in ComBat batch-corrected gene expression data. 21

Supplementary Methods

Study eligibility criteria

1. Patient is ≥18 years of age upon enrolment
2. Patient is willing to provide specimens (sputum, urine, blood, stool, buccal, and peridontal swab)
3. Patient has signed informed consent form
4. Patient is suspected of having TB based on symptoms, or is HIV-positive and has had contact with a TB case
    a. HIV negative patients require any one of the following TB symptoms:
        i. Cough ≥ two weeks
        ii. Drenching night sweats
        iii. Fever
        iv. Weight loss in last 1-8weeks
    b. HIV positive patients need to be in contact with a confirmed TB patient, or require any one of the following TB symptoms:
        i. Cough for any duration
        ii. Drenching night sweats
        iii. Fever
        iv. Weight loss in last 1-8weeks

TB symptom score

The modified TB symptom score (TBscoreII), encompassed cough, chest pain, body mass index, haemoglobin levels, and mid upper arm circumference.[1] A higher symptom score reflected more severe disease.

Sputum culture

Of two sputa provided upon patient enrolment, the sputum that appeared more viscous and is hence more likely to be culture-positive,[2] was used for smear microscopy and liquid culture (the other sputum was used for Xpert MTB/RIF). Cultures overgrown with acid-fast-negative bacteria were classified as contaminated and excluded. MTBDR*plus* was done on isolates and used for *Mycobacterium tuberculosis* complex, rifampicin and isoniazid susceptibility detection.

Batch correction of RNA sequencing data

We determined the need for, and effect of, batch correction by principal component analysis (PCA), using the *prcomp* function in R. PCA of blood RNA sequencing data from the entire transcriptome (n=20177 genes) showed clear separation of samples into two clusters in the first principal component, which were not explained by any biological or known technical variables (Figure S1). This separation remained evident in a PCA of all samples, when including only the 25% least variable genes (n=5044) among samples of the larger observed cluster (Figure S2A). To account for this observation, we tested two batch correction techniques, using the *ComBat* and *sva* functions from the sva package in R, respectively.[3] For *ComBat*, we specified the two observed clusters as the batch effect requiring correction, whilst surrogate variable analysis (SVA) estimated unwanted

variation directly from the gene expression data.[3] Biological differences of interest (TB status, HIV status, age, sex, and ethnicity) were included in the SVA model and were hence protected from removal as sources of unwanted variation. SVA identified 13 surrogate variables. Repeat PCA post batch correction with either technique showed homogenous sample distributions, irrespective of initial clustering (Figure S2B-C). Since SVA preserved the specified outcomes of interest whilst correcting any other, unwanted variation, and because samples clustered more tightly after batch correction with SVA, we used SVA-adjusted data for the primary downstream analyses.

As SVA estimates unwanted batch variation directly from the gene expression data, it carries a risk of over-fitting. Although known biological differences of interest were included in the SVA model, diagnostic performance of transcriptional signatures might be slightly over-estimated in this analysis. In a sensitivity analysis, we therefore re-analyzed the data using *ComBat* as batch correction method, where neither disease outcome nor other co-variates were specified. This produced reassuringly similar results, although with slightly lower overall AUROCs (Figure S8), which may reflect some residual batch effect.

Eligibility criteria for candidate signatures identified through systematic review (adapted from Gupta et al [4])

- Whole blood mRNA signature discovered with primary objective of diagnosis of active or incipient TB in humans.
- Approach to discovery reduces dimensionality of the signature by a defined approach to feature selection to ensure that signature is 'concise' and may therefore be more amenable to clinical translation.
- Availability of gene names that comprise signature, along with corresponding equation or modelling approach.
- Signature (including component genes, and modelling approach) validated in at least one independent 'test' set that is distinct from the training set, to prioritize inclusion of signatures discovered in higher quality studies, and in order to enable reliable signature reconstruction.
- Where multiple signatures were discovered for the same intended purpose and from the same training dataset, that with greatest accuracy in the validation set will be included. Where accuracy was equivalent (as defined by the area under the ROC curve), we will include the most parsimonious signature.

Calculation of signature scores

Constituent genes (gene symbols) of the original signatures were updated to current nomenclature to match gene symbol annotation in the current RNAseq dataset. Genes that were not present in the RNAseq data were omitted from score calculations. This included signature genes whose annotations have been withdrawn, and non-coding genes that were not part of our protein-coding RNAseq data matrix. All gene changes are summarized in appendix 2. Unless otherwise stated, log2-transformed transcripts per million (TPM) values were used for score calculations.

Disease risk score

The disease risk score is calculated as difference of sums between genes that, in the original publication, were found to be up-regulated and those that were down-regulated in TB. The diseases risk score was used for

Anderson and Kaforou signatures.[5,6] The original signatures were based on Illumina probe IDs and resulted in gene symbol duplicates. Duplicates were retained (and therefore counted twice) in the current calculations.

Modified disease risk score

The modified disease risk score as defined by Singhania et al. is based on counts per million (CPM) values and, following trimmed mean of M-values (TMM) normalization, is calculated as sum of non-log transformed expression levels.[7] In the current dataset, log2-transformed CPM values were used as input for TMM normalization with the epigenomix package in R. TMM-normalized CPM values were then batch-corrected by surrogate variable analysis, and the modified disease risk score calculated as sum of signature genes' exponents.

Difference of means

The Penn-Nicholson6 signature was calculated as difference of means,[8] using the following formula: mean (GBP2, FCGR1B, SERPING1) – mean (TUBGCP6, TRMT2A, SDR39U1).

Sum of standardized expression

To calculate the Qian17 signature,[9] log2 TPM values were first standardized by subtracting the mean and dividing by the standard deviation of the gene expression values across all samples. Standardized gene expression values were then summed.

Unsigned sums

For the Rajan5 signature,[10] the log2 TPM values of the five constituent genes were summed, independent of whether they had been found to be up- or down-regulated in TB.

LASSO regression

The Gjoen8 signature was calculated as sum of weighted gene expression exponents, using the regression coefficients from the original publication.[11] The original 7-transcript signature includes IFITM1/3 as single transcript because the probes used for discovery could not separate the two genes.[11] Here, we included both IFITM1 and IFITM3, with equal weighting to both genes.

Random forest

The Maertzdorf4 random forest model was constructed with the randomForest package in R, using the original training data from Maertzdorf et al.[12] To calculate the Maertzdorf4 signature in the current RNAseq data, log2 TPM values were first standardized by subtracting the mean and dividing by the standard deviation of the gene expression values across all samples. The *predict* function in the randomForest package was then used to obtain Maertzdorf4 signature scores.

Support vector machines (SVM)

All SVM models were constructed with linear kernel in the *ksvm* function of the kernlab package in R, using the original training data for the respective signature. Signature scores were then obtained with the *predict* function of the kernlab package. The signatures from Walter et al. were based on Affymetrix probe IDs and resulted in

gene symbol duplicates,[13] which were removed for model training, retaining only the gene symbol with highest average expression across all samples in the training set.

The Duffy10 signature was discovered as multinomial random forest model.[14] Our attempts to reconstruct this model following the authors' methods resulted in an inferior performance in a common test set (our AUROC=0·81 vs. original AUROC=0·88). We therefore used the ten signature genes in a binary SVM model, which achieved a better performance in the same test set (AUROC=0·84) and was included in the final analysis.

Pair ratio algorithm

The modelling approach for the 4-gene signature by Suliman et al. was not clear from the original description.[15] We recreated this using two approaches: (1) as difference of sums ((GAS6 + SEPT4) - (CD1C + BLK)); and (2) as SVM model using the four constituent gene pairs, as previously described.[16] Since in a common test set (Table S1), the former approach achieved marginally better performance (difference of sums, AUROC=0·66 vs. SVM, AUROC=0·65), that was closer to the authors' original description (AUROC=0·67), this was included in the final analysis.

Diagnostic algorithm combining sputum Ultra analysis with blood transcriptional signatures

Sputum Ultra analysis returns more false-positives (culture-negatives) compared to Xpert, resulting in reduced specificity, especially among patients with previous TB disease and those with Ultra trace results.[18] To test whether Ultra specificity could be improved, we compared performance of a diagnostic algorithm that combined Ultra analysis with blood transcriptional signatures to that of Ultra analysis alone. Sensitivity and specificity were calculated among 154 patients with Ultra results, before and after re-classification of selected Ultra-positive tests by transcriptional signatures. Re-classification was based on the original maximum Youden index threshold of each signature as cut-off for signature positivity, and was restricted to either all Ultra-positive results (n=51), patients with Ultra trace results (n=9), or patients with Ultra-positive results and a history of TB disease (n=21).

Best-case four-culture simulation

One sputum Mycobacteria Growth Indicator Tube 960 (MGIT960) culture has a sensitivity of 84% for smear-negative TB, compared to a composite reference standard of four cultures (two MGIT960 and two Löwenstein–Jensen) and three smears (Ziehl–Neelsen).[17] While ideal, comprehensive reference standards are often unfeasible in resource-limited HIV-endemic settings for reasons of cost, logistics, and sputum-scarce TB. Therefore, in a scenario permitting us to be as generous as possible to the performance of transcriptional signatures, we assessed what the most optimistic effect of additional cultures would be on signature performance. To do this, we assumed that all smear-negative patients potentially missed by a single MGIT960, and hence originally classified as non-TB, scored above the maximum Youden index of each transcriptional signature. For example, for Sweeney3 there were initially 34 smear-negative, culture-positive TB patients with a signature score above the Youden index. We adjusted this to 40 (=34/0.84), thereby re-classifying six initial 'false-positives' to 'true-positives' (provided there were sufficient smear-negative patients who were initially 'false-positive'), and recalculating sensitivity and specificity at the original Youden index threshold.

References

1 Rudolf F, Lemvik G, Abate E, *et al.* TBscore II: Refining and validating a simple clinical score for treatment monitoring of patients with pulmonary tuberculosis. *Scand J Infect Dis* 2013; **45**: 825–36.

2 Blakemore R, Nabeta P, Davidow AL, *et al.* A Multisite Assessment of the Quantitative Capabilities of the Xpert MTB/RIF Assay. *Am J Respir Crit Care Med* 2011; **184**: 1076–84.

3 Leek JT, Johnson WE, Parker HS, Jaffe AE, Storey JD. The sva package for removing batch effects and other unwanted variation in high-throughput experiments. *Bioinformatics* 2012; **28**: 882–3.

4 Gupta RK, Turner CT, Venturini C, *et al.* Concise whole blood transcriptional signatures for incipient tuberculosis: a systematic review and patient-level pooled meta-analysis. *Lancet Respir Med* 2020; **0**. DOI:10.1016/s2213-2600(19)30282-6.

5 Anderson ST, Kaforou M, Brent AJ, *et al.* Diagnosis of childhood tuberculosis and host RNA expression in Africa. *N Engl J Med* 2014; **370**: 1712–23.

6 Kaforou M, Wright VJ, Oni T, *et al.* Detection of tuberculosis in HIV-infected and -uninfected African adults using whole blood RNA expression signatures: a case-control study. *PLoS Med* 2013; **10**: e1001538.

7 Singhania A, Verma R, Graham CM, *et al.* A modular transcriptional signature identifies phenotypic heterogeneity of human tuberculosis infection. *Nat Commun* 2018; **9**: 2308.

8 Penn-Nicholson A, Mbandi SK, Thompson E, *et al.* RISK6, a universal 6-gene transcriptomic signature of TB disease risk, diagnosis and treatment response. *medRxiv* 2019; : 19006197.

9 Qian Z, Lv J, Kelly GT, *et al.* Expression of nuclear factor, erythroid 2-like 2-mediated genes differentiates tuberculosis. *Tuberculosis (Edinb)* 2016; **99**: 56–62.

10 Rajan J, Deng X, Seielstad M, *et al.* Performance of gene expression signatures in the context of intensified tuberculosis case finding among people living with HIV (PLHIV). *Am J Respir Crit Care Med* 2017; **195**. DOI:http://dx.doi.org/10.1164/ajrccm-conference.2017.A26.

11 Gjoen JE, Jenum S, Sivakumaran D, *et al.* Novel transcriptional signatures for sputum-independent diagnostics of tuberculosis in children. *Sci Rep* 2017; **7**: 5839.

12 Maertzdorf J, McEwen G, Weiner 3rd J, *et al.* Concise gene signature for point-of-care classification of tuberculosis. *EMBO Mol Med* 2016; **8**: 86–95.

13 Walter ND, Miller MA, Vasquez J, *et al.* Blood Transcriptional Biomarkers for Active Tuberculosis among Patients in the United States: a Case-Control Study with Systematic Cross-Classifier Evaluation. *J Clin Microbiol* 2016; **54**: 274–82.

14 Duffy FJ, Thompson EG, Scriba TJ, Zak DE. Multinomial modelling of TB/HIV co-infection yields a robust predictive signature and generates hypotheses about the HIV+TB+ disease state. *PLoS One* 2019;

14: e0219322.

15 Suliman S, Thompson EG, Sutherland J, *et al.* Four-gene pan-African blood signature predicts progression to tuberculosis. *Am J Respir Crit Care Med* 2018; **197**: 1198–208.

16 Warsinske H, Vashisht R, Khatri P. Host-response-based gene signatures for tuberculosis diagnosis: A systematic comparison of 16 signatures. *PLOS Med* 2019; **16**: e1002786.

17 Boehme CC, Nabeta P, Hillemann D, *et al.* Rapid Molecular Detection of Tuberculosis and Rifampin Resistance. *N Engl J Med* 2010; **363**: 1005–15.

18 Dorman SE, Schumacher SG, Alland D, *et al.* Xpert MTB/RIF Ultra for detection of Mycobacterium tuberculosis and rifampicin resistance: a prospective multicentre diagnostic accuracy study. *Lancet Infect Dis* 2018; **18**: 76–84.

Supplementary Tables

Table S1. Signature validation against the authors' original descriptions by comparing area under the receiver operating characteristic curve (AUROC) in common datasets.

Signature	Original AUROC	Our AUROC (95% CI)	Common dataset (GEO accession number)
Anderson39.LTBI	1·00	0·99 (0·98 - 1·00)	Anderson validation (GSE39941, Kenya subset)
Anderson39.OD *	0·89	0·74 (0·63 - 0·85)	Anderson validation (GSE39941, Kenya subset)
Duffy10	0·88	0·84 (0·78 - 0·89)	Kaforou validation (GSE37250, Malawi subset)
Kaforou25	0·99	0·99 (0·97 - 1·00)	Berry validation (GSE19442)
Kaforou39	1·00	0·99 (0·97 - 1·00)	Berry validation (GSE19491)
Maertzdorf4	0·98	1·00 (1·00 - 1·00)	Maertzdorf training (GSE74092)
Penn-Nicholson6	0·96	0·95 (0·92 - 0·98)	Kaforou validation (GSE37250, HIV-negative subset)
Suliman4	0·67	0·66 (0·55 - 0·77)	Suliman test (GSE94438)
Walter46	0·98	0·98 (0·94 - 1·00)	Walter test (GSE73408)
Walter32	0·90	0·92 (0·81 - 1·00)	Walter test (GSE73408)
Walter101	0·94	0·96 (0·90 - 1·00)	Walter test (GSE73408)
Zak16	0·69	0·71 (0·56 - 0·85)	Zak test (GSE79362)

Signatures were validated if the model had to be reconstructed or if the number of included genes changed because not all genes were present in the current RNAseq data. No validation was possible for the Huang11 and Kaforou45 signatures as no AUCs were reported by the authors in their original dataset. * The Anderson39.OD signature had originally 51 genes, but only 39 genes were present in our protein-coding RNAseq dataset, driving the decreased accuracy of our model. LTBI = latent tuberculosis infection; OD = other diseases; CI = confidence interval; GEO = Gene Expression Omnibus (https://www.ncbi.nlm.nih.gov/geo/); HIV = human immunodeficiency virus.

Table S2. Baseline characteristics of patients included in and excluded from the study subset.

	**Study subset**	**No blood RNA sample collected**	**No reference or index test data**
Patients	181 (60)	97	24
Age, years	35 (27 – 48)	40 (27 – 50)	33 (25 – 39)
Male sex	94 (52)	51 (53)	13 (54)
Ethnicity			
Black	28 (15)	7 (7)	4 (17)
Mixed ancestry	153 (85)	90 (93)	20 (83)
HIV status			
Unknown	1 (1)	--	--
Uninfected	136 (75)	81 (84)	20 (83)
Infected	44 (24)	16 (16)	4 (17)
Anti-retroviral therapy *			
No	24 (55)	3 (19)	2 (50)
Yes	15 (34)	13 (81)	2 (50)
Unknown ‡	5 (11)	--	--
CD4 count *, cells/µl	334 (192 – 606)	316 (194 – 498)	270 (187 – 404)
Haemoglobin, g/dl	13·7 (12·4 – 14·8)	13·5 (12·2 - 15)	14·1 (12·8 - 15·5)
Leucocytes, 10^9 cells/l	8 (6·1 – 10·2)	8·4 (6·7 - 11·3)	6·4 (5·2 - 8·1)
Body mass index, kg/m^2	19·9 (17·8 – 22·5)	20·3 (17·9 -23·7)	20·4 (19 - 22·6)
TB symptom score	2 (2 – 3)	2 (1 – 3)	2 (1 – 3)
Previous TB			
No	115 (64)	51 (53)	20 (83)
Yes	66 (36)	46 (47)	4 (17)
Liquid culture			
Positive	53 (29)	24 (25)	3 (13)
Negative	128 (71)	61 (63)	11 (46)
No result	--	12 (12)	10 (42)
Sputum smear			
Positive	15 (8)	17 (18)	2 (8)
Negative	157 (87)	78 (80)	15 (63)
Not done	9 (5)	2 (2)	7 (29)
Xpert MTB/RIF			
Positive	44 (24)	23 (24)	2 (8)
Negative	134 (74)	72 (74)	22 (92)
No result	2 (2)	2 (2)	--
Not done	1 (1)	--	--
Ultra			
Positive †	51 (28)	29 (30)	3 (13)
Negative	103 (57)	61 (63)	11 (46)
No result	10 (6)	4 (4)	3 (13)
Not done	17 (9)	3 (3)	7 (29)

Data are n (%) or median (interquartile range). TB = tuberculosis; HIV = human immunodeficiency virus. * Anti-retroviral therapy and CD4 counts amongst HIV-infected patients only. † Ultra-positive results include tests where traces of *Mycobacterium tuberculosis* were detected. Missing data "Study subset": CD4 (n=1), haemoglobin (n=3), leucocytes (n=3), body mass index (n=1), symptom score (n=3). Missing data "No blood RNA sample collected": CD4 (n=1), haemoglobin (n=6), leucocytes (n=6), body mass index (n=2), symptom score (n=7). Missing data "No reference or index test data": CD4 (n=1).

Table S3. Area under the receiver operating characteristic curve (AUROC) of candidate transcriptional signatures for tuberculosis.

Signature	AUROC, % (95% CI)	p-value
Sweeney3	90·6 (85·6 - 95·6)	ref
Kaforou25	86·9 (80·9 - 92·9)	0·21
Roe3	86·9 (80·3 - 93·5)	0·11
BATF2	86·8 (80·6 - 93·1)	0·10
Penn-Nicholson6	84·5 (77·9 - 91)	0·0382
Suliman2	83·9 (77·1 - 90·7)	0·0357
Gliddon3	82·7 (75·6 - 89·8)	0·0326
Maertzdorf4	81·9 (75·2 - 88·5)	0·0028
Kaforou39	81·6 (75 - 88·3)	0·0011
Duffy10	81 (74·3 - 87·8)	0·0066
Zak16	79·8 (72·4 - 87·2)	0·0003
Anderson39.OD	79·5 (72·5 - 86·4)	0·0002
NPC2	79·4 (72·5 - 86·3)	0·0006
Qian17	78·2 (70·6 - 85·9)	0·0003
Gliddon4	74·3 (66·5 - 82·2)	<0·0001
Rajan5	72·9 (63·8 - 81·9)	<0·0001
Roe5	71·5 (62·7 - 80·3)	<0·0001
Suliman4	71 (62·4 - 79·5)	<0·0001
Kaforou45	69·6 (61·4 - 77·9)	<0·0001
Singhania20	69·2 (61·3 - 77·2)	<0·0001
Huang11	67·4 (58·5 - 76·3)	<0·0001
Walter101	65·7 (57·2 - 74·1)	<0·0001
Anderson39.LTBI	60·8 (51·8 - 69·8)	<0·0001
Walter32	59 (50·2 - 67·7)	<0·0001
Gjoen8	57·2 (48·2 - 66·2)	<0·0001
Roe4	56·7 (47·5 - 65·8)	<0·0001
Walter46	50·1 (40·5 - 59·7)	<0·0001

Signature scores were calculated following surrogate variable analysis of gene expression data. P values represent paired comparisons against the best performing signature (Sweeney3), using DeLong tests. CI = confidence interval.

Table S4. Area under the receiver operating characteristic curve (AUROC) of the four best-performing blood transcriptional signatures in subgroup analyses.

	non-TB n (%)	TB n (%)	BATF2 AUROC, % (95% CI)	Kaforou25 AUROC, % (95% CI)	Roe3 AUROC, % (95% CI)	Sweeney3 AUROC, % (95% CI)
**Age**						
< 35 years	55 (65)	30 (35)	88·1 (79·8 - 96·4)	90·3 (83·2 - 97·4)	87·8 (79·2 - 96·3)	93·3 (88·5 - 98·2)
≥ 35 years	72 (75)	24 (25)	85·8 (76·3 - 95·2)	83·6 (73·9 - 93·3)	86·1 (75·7 - 96·5)	88·1 (78·7 - 97·6)
p-value			0·71	0·28	0·81	0·34
**Sex**						
Female	61 (70)	26 (30)	85·3 (76·5 - 94·1)	87·6 (79·8 - 95·3)	84·7 (75 - 94·3)	90·5 (84·2 - 96·9)
Male	66 (70)	28 (30)	87·7 (78·5 - 96·9)	85·6 (76 - 95·2)	88 (78·5 - 97·6)	90·3 (82·2 - 98·4)
p-value			0·72	0·75	0·63	0·97
**Ethnicity**						
Mixed ancestry	113 (74)	40 (26)	91·7 (86·4 - 97)	92·1 (87 - 97·3)	91·3 (85·6 - 96·9)	93·8 (90·3 - 97·3)
Black	14 (50)	14 (50)	72·4 (52·9 - 92)	66·8 (45·7 - 88)	73·5 (53·6 - 93·3)	80·6 (63·1 - 98·1)
p-value			0·07	**0·030**	0·10	0·16
**HIV status**						
Infected	27 (61)	17 (39)	91·3 (83·2 - 99·3)	85 (73·3 - 96·6)	89·3 (78·7 - 99·9)	94·6 (88·6 - 100)
Uninfected	99 (73)	37 (27)	84·7 (76·3 - 93)	87·6 (80·1 - 95)	85·1 (76·4 - 93·8)	88·6 (81·9 - 95·3)
p-value			0·26	0·71	0·55	0·19
**Haemoglobin**						
< 12·5 g/dl	21 (44)	27 (56)	87·7 (78 - 97·3)	86·2 (75·9 - 96·6)	86·4 (75·7 - 97·1)	88·4 (79·1 - 97·7)
≥ 12·5 g/dl	103 (79)	27 (21)	83·4 (73·3 - 93·6)	85 (76·1 - 93·9)	84·8 (74·6 - 95·1)	87·7 (78·9 - 96·5)
p-value			0·55	0·86	0·83	0·92
**Body mass index**						
< 19 kg/m^2	40 (60)	27 (40)	92·9 (86·5 - 99·3)	88·1 (79·7 - 96·6)	93·6 (86·7 - 100)	97·5 (94·6 - 100)
>= 19 kg/m^2	86 (76)	27 (24)	82·6 (72·1 - 93)	85·1 (76 - 94·1)	81·8 (70·9 - 92·7)	86·2 (77·3 - 95·1)
p-value			0·10	0·62	0·08	**0·020**
**Previous TB**						
No	81 (70)	34 (30)	86·9 (79·2 - 94·7)	86·5 (79 - 94)	88·3 (80·7 - 95·9)	92 (86·4 - 97·6)
Yes	46 (70)	20 (30)	86·2 (74·7 - 97·7)	88·6 (78·9 - 98·3)	85·1 (72·7 - 97·5)	88·4 (78·9 - 97·9)
p-value			0·92	0·74	0·67	0·52
**Sputum smear***						
Negative	127 (77)	37 (23)	85·8 (78·4 - 93·3)	84·1 (76·2 - 91·9)	86·3 (78·4 - 94·3)	90·4 (84·2 - 96·5)
Positive	127 (90)	14 (10)	88·8 (76·1 - 100)	92·4 (85·8 - 98·9)	88·1 (74·9 - 100)	91·2 (82·7 - 99·6)
p-value			0·69	0·11	0·82	0·88
**Symptom score**						
< 3	85 (79)	23 (21)	81·2 (69·2 - 93·2)	82·4 (71·9 - 92·8)	82·1 (69·9 - 94·3)	86·1 (75·8 - 96·4)
≥ 3	39 (56)	31 (44)	91·6 (85·2 - 98·1)	90·8 (83·8 - 97·8)	91·1 (83·9 - 98·4)	94·2 (89 - 99·4)
p-value			0·14	0·19	0·21	0·17

Continuous variables were classified into binary categories using a value close to the median of the tuberculosis (TB) group to separate the categories. CI = confidence interval; HIV = human immunodeficiency virus. * Sputum smear status was differentiated for TB patients only; all non-TB patients were included irrespective of smear status. P values compare AUROCs of the same signature in opposite subgroups, using DeLong tests.

Table S5. Diagnostic performance metrics at the maximum Youden index in a best-case four-culture simulation analysis.

	Re-classified patients, n	Non-TB, n	TB, n	Sensitivity, % (95% CI)	Specificity, % (95% CI)	PPV, % (95% CI)	NPV, % (95% CI)
BATF2	6	121	60	88·3 (77·8 - 94·2)	83·5 (75·8 - 89)	72·6 (61·4 - 81·5)	93·5 (87·2 - 96·8)
Kaforou25	5	122	59	76·3 (64 - 85·3)	93·4 (87·6 - 96·6)	84·9 (72·9 - 92·1)	89·1 (82·5 - 93·4)
Roe3	6	121	60	91·7 (81·9 - 96·4)	77·7 (69·5 - 84·2)	67·1 (56·3 - 76·3)	94·9 (88·7 - 97·8)
Sweeney3	6	121	60	88·3 (77·8 - 94·2)	89·3 (82·5 - 93·6)	80·3 (69·2 - 88·1)	93·9 (88 - 97)

The maximum permissible number of smear-negative, culture-negative patients with signature scores above the maximum Youden index threshold were re-classified as tuberculosis (TB), based on the estimated sensitivity of a single liquid culture compared to a four-culture reference. Performance metrics were then calculated in the full dataset of 181 patients. CI = confidence interval; PPV = positive predictive value; NPV = negative predictive value.

Supplementary Figures

Figure S1. Estimates of sample size and power. (A.) Total number of participants needed to achieve 90% sensitivity and 70% specificity by disease prevalence with a 10% margin of error. **(B.)** Total number of participants required for 80% power to detect statistically significant reductions by disease prevalence stratified by the difference of area under receiver operator characteristic curves (Δ AUROC) compared to a reference AUROC of 0.9. Dashed lines represent sample size of 181 in the present study.

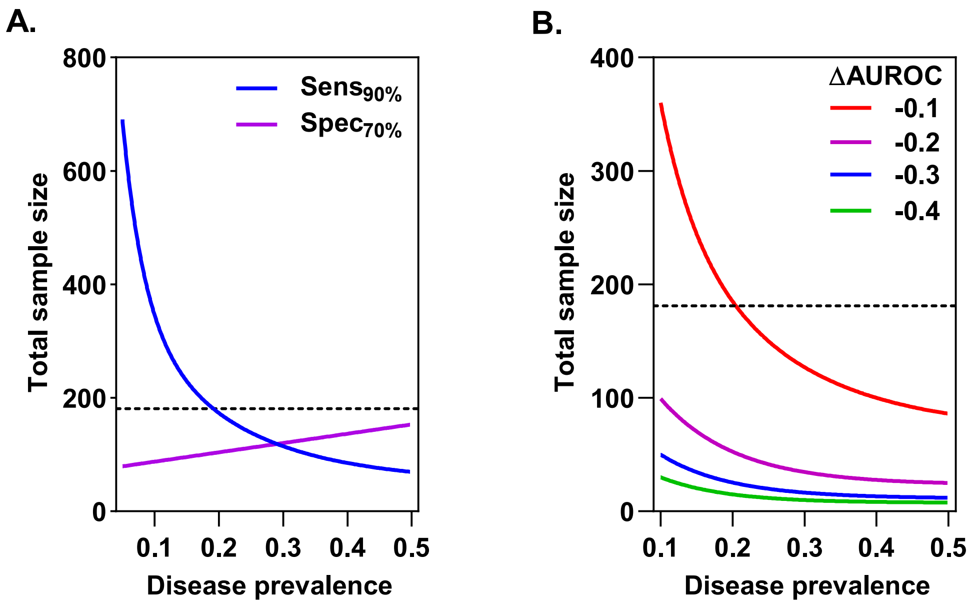

Figure S2. Principal component analysis of gene expression data shows separation of samples along the first principal component (A.) which is not explained by biological (B.) or known technical variables (C.) TB = tuberculosis; HIV = human immunodeficiency virus.

A.

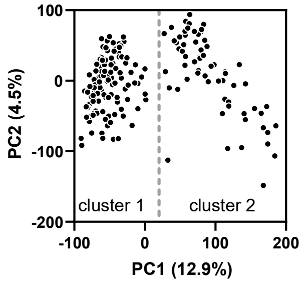

B.

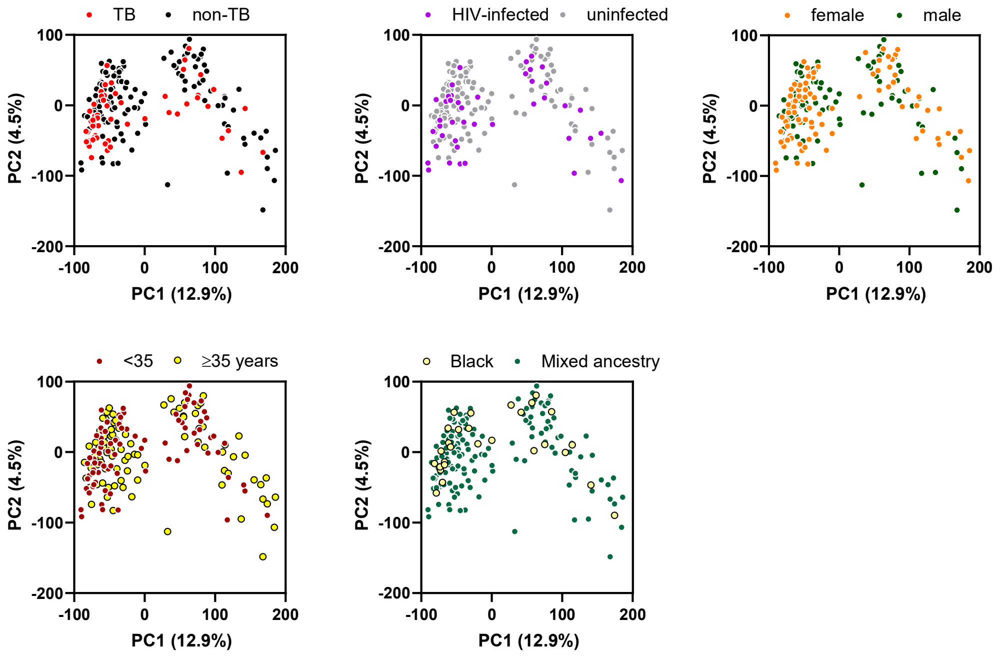

C.

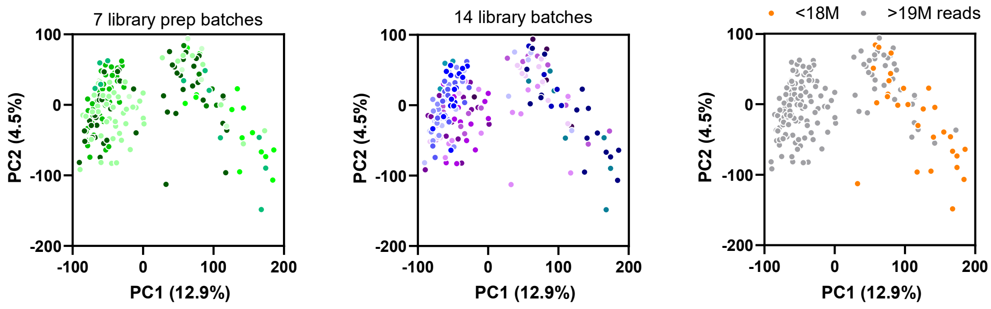

Figure S3. Principal component analysis (PCA) of unadjusted gene expression data, and after batch correction by surrogate variable analysis (SVA) or ComBat. PCA was performed with (**A.**) all genes or (**B.**) the least variable genes amongst samples belonging to bigger cluster (cluster 1; blue dots).

A.

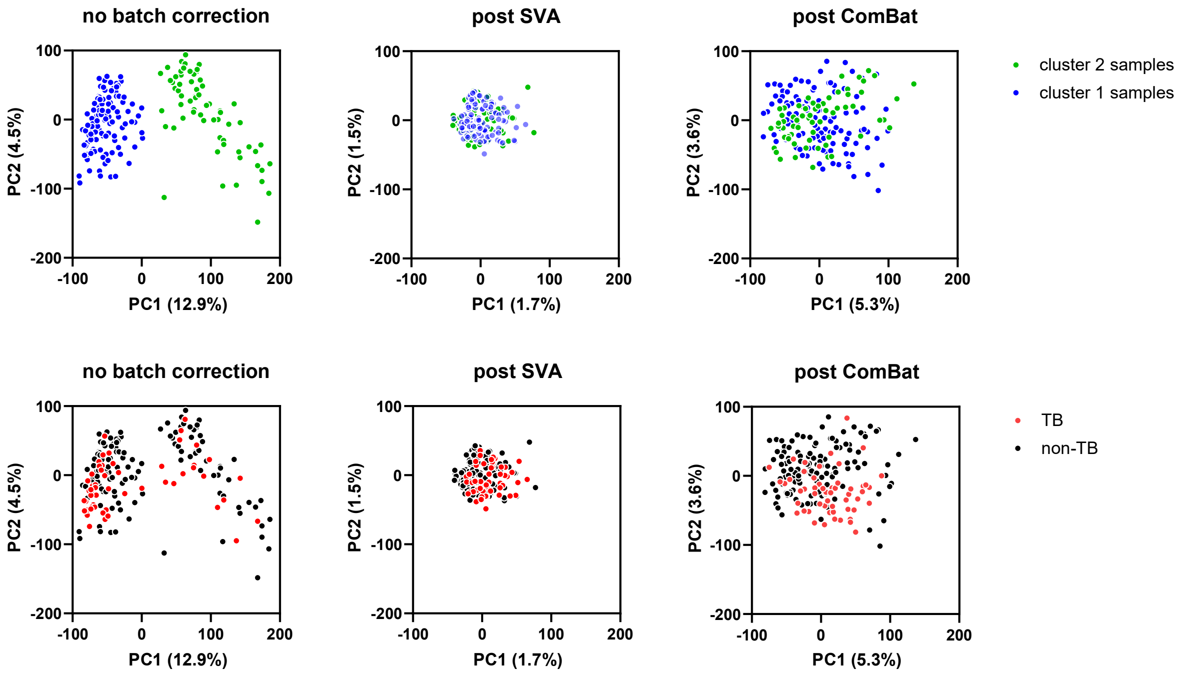

B.

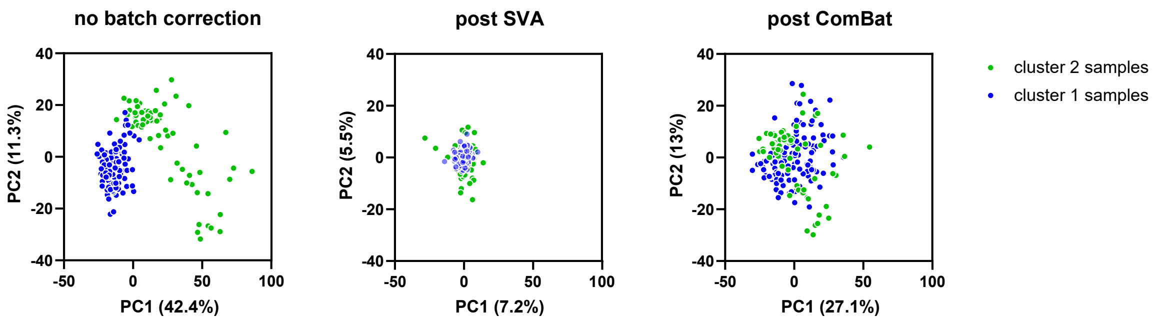

Figure S4. Venn diagram visualizing concordance between culture-positivity, Xpert-positivity and initiation of tuberculosis (TB) treatment.

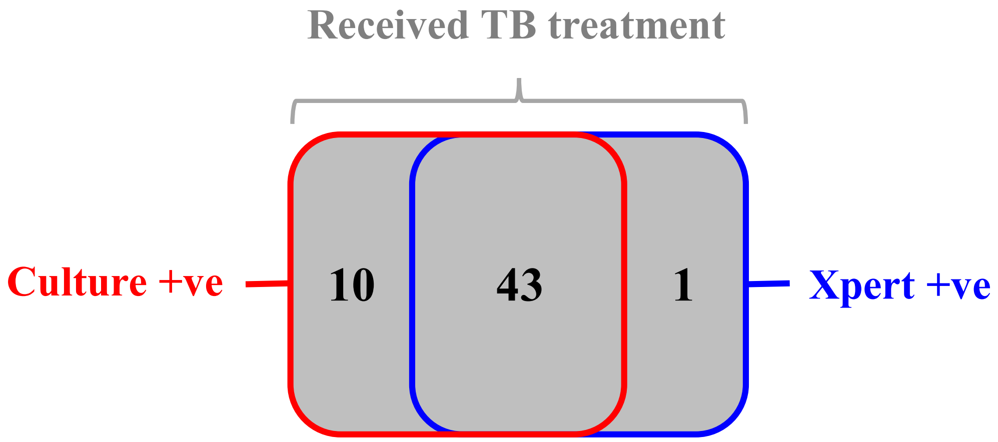

Figure S5. Receiver operating characteristic (ROC) curves of clinical characteristics, benchmarked against World Health Organization Target Product Profile criteria for a tuberculosis triage test. Minimum target criteria (90% sensitivity, 70% specificity) are indicated by the dashed black box, optimum criteria (95% sensitivity, 80% specificity) are indicated by the blue box. The light blue shaded area represents the 95% confidence intervals of the ROC curves. Area under the ROC curve values are indicated with 95% confidence intervals in brackets.

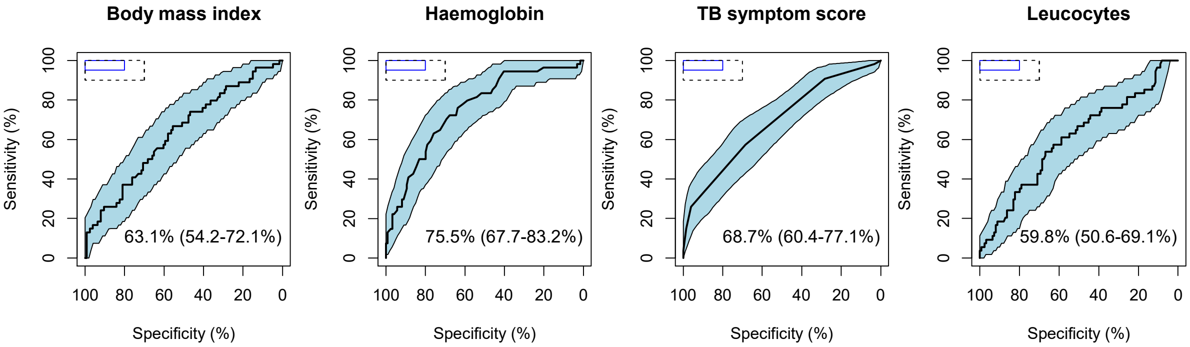

Figure S6. Flowchart showing systematic review process to identify concise whole blood transcriptional signatures for active or incipient tuberculosis. The systematic search was performed on 15/04/2019. Two articles that were published after this date were included following expert consultation. All screened articles are listed in appendix 3, with reviewed full texts matched against inclusion criteria.

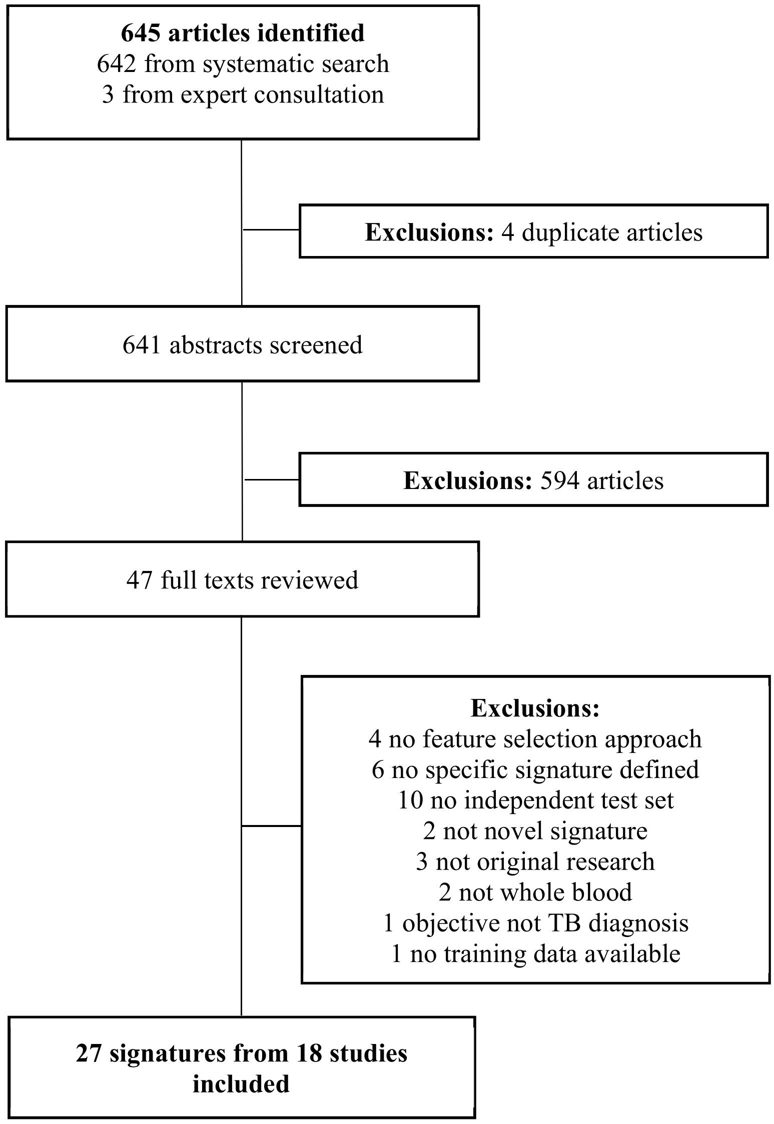

Figure S7. Tuberculosis (TB) signature scores do not correlate with bacterial load or symptom duration. Spearman correlation analysis was restricted to patients with culture-positive TB (n=53; **A.**), Xpert-positive TB (n=44; **B.**), or culture- or Xpert-positive TB (n=54; **C.**).

A.

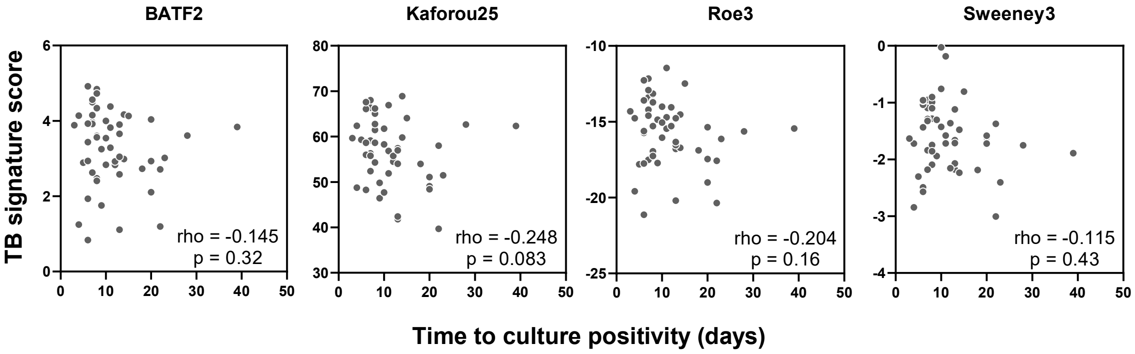

B.

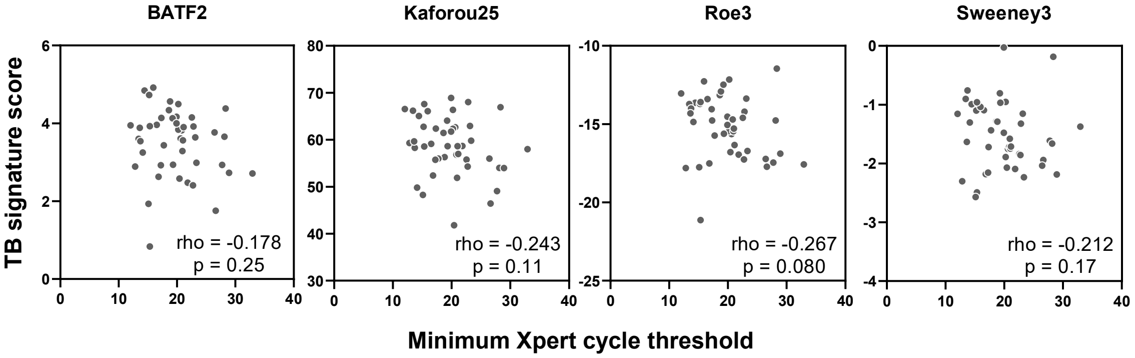

C.

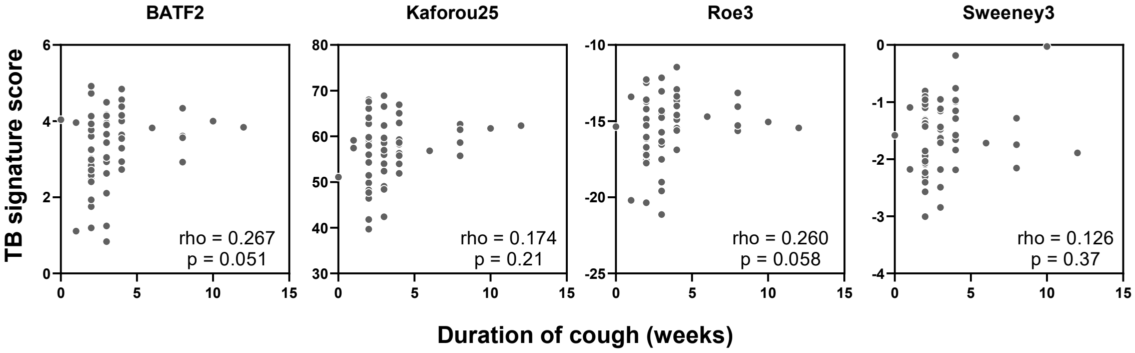

Figure S8. Sensitivity analysis with tuberculosis (TB) case definition restricted to culture-proven patients. Diagnostic accuracy of the four best-performing signatures was assessed among 53 patients with liquid culture-positive TB and 128 culture-negative non-TB patients. **A.** Paired comparisons of area under the receiver operating characteristic curves (AUROCs) against the best-performing signature (Sweeney3). **B.** Paired comparisons of ROC curves between HIV-infected and HIV-uninfected patients. Shaded areas represent the 95% confidence interval of the ROC curves. AUROC values are indicated with 95% confidence intervals in brackets. P values are derived from DeLong tests. **C.** ROC curves benchmarked against minimum (dashed black box) and optimum (blue box) target criteria for a TB triage test. Blue shaded area represents the 95% confidence interval of the ROC curve. AUROC values are indicated with 95% confidence intervals in brackets. **D.** Diagnostic performance metrics at different thresholds. CI = confidence interval; HIV = human immunodeficiency virus; NPV = negative predictive value; PPV = positive predictive value.

A.

Signature	AUROC, % (95% CI)	p-value
Sweeney3	90·6 (85·5 - 95·6)	ref
Roe3	86·9 (80·2 - 93·6)	0·12
Kaforou25	86·6 (80·5 - 92·7)	0·18
BATF2	86·3 (79·9 - 92·7)	0·06

B.

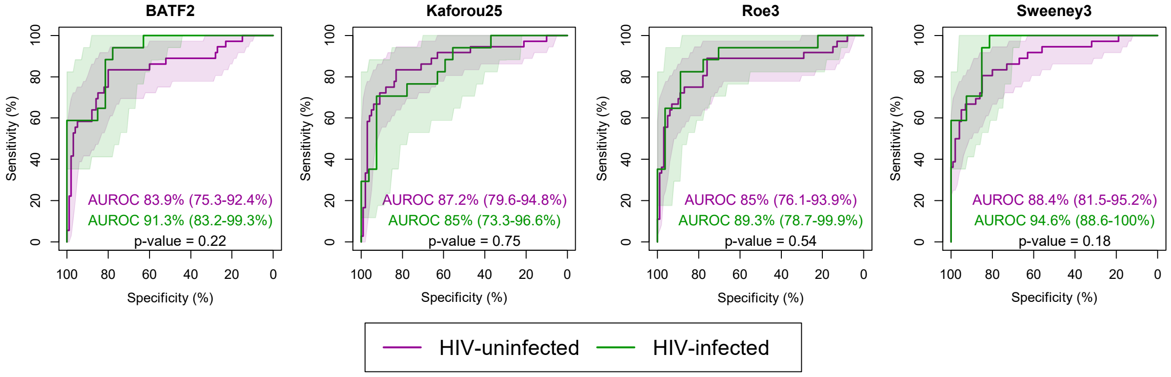

C.

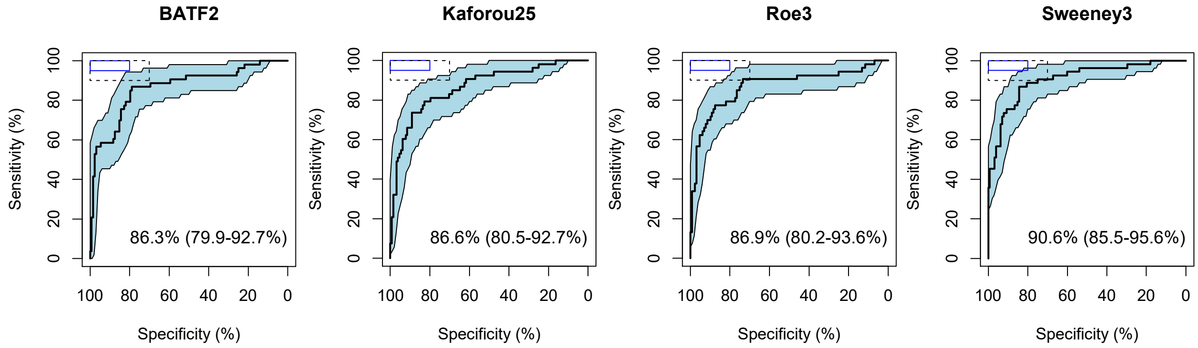

D.

	Sensitivity, % (95% CI)	Specificity, % (95% CI)	PPV, % (95% CI)	NPV, % (95% CI)
**At maximum Youden index**				
BATF2	86·8 (75·2 - 93·5)	78·9 (71 - 85·1)	63 (51·5 - 73·2)	93·5 (87·2 - 96·8)
Kaforou25	73·6 (60·4 - 83·6)	89·1 (82·5 - 93·4)	73·6 (60·4 - 83·6)	89·1 (82·5 - 93·4)
Roe3	77·4 (64·5 - 86·5)	87·5 (80·7 - 92·2)	71·9 (59·2 - 81·9)	90·3 (83·8 - 94·4)
Sweeney3	86·8 (75·2 - 93·5)	84·4 (77·1 - 89·7)	69·7 (57·8 - 79·4)	93·9 (88 - 97)
**At minimum sensitivity for a triage test**				
BATF2	90	59·4 (50·7 - 67·5)	47·8 (38·3 - 57·5)	93·5 (85·9 - 97·1)
Kaforou25		61·7 (53·1 - 69·7)	49·3 (39·6 - 59·1)	93·7 (86·4 - 97·2)
Roe3		73·4 (65·2 - 80·3)	58·4 (47·6 - 68·5)	94·7 (88·4 - 97·6)
Sweeney3		75 (66·8 - 81·7)	59·8 (48·9 - 69·9)	94·8 (88·6 - 97·7)
**At minimum specificity for a triage test**				
BATF2	86·8 (75·2 - 93·5)	70	54·5 (43·9 - 64·7)	92·8 (85·8 - 96·4)
Kaforou25	83 (70·8 - 90·8)		53·4 (42·7 - 63·8)	90·9 (83·6 - 95·1)
Roe3	90·6 (79·7 - 95·9)		55·6 (45·1 - 65·6)	94·7 (88·2 - 97·7)
Sweeney3	90·6 (79·7 - 95·9)		55·6 (45·1 - 65·6)	94·7 (88·2 - 97·7)
**At optimum sensitivity for a triage test**				
BATF2	95	25 (18·3 - 33·2)	34·4 (27·2 - 42·4)	92·4 (78·7 - 97·5)
Kaforou25		28·1 (21·1 - 36·5)	35·4 (28 - 43·5)	93·1 (80·7 - 97·8)
Roe3		13·3 (8·5 - 20·2)	31·2 (24·6 - 38·7)	86·5 (65·5 - 95·6)
Sweeney3		53·9 (45·3 - 62·3)	46 (37 - 55·4)	96·3 (89·1 - 98·8)
**At optimum specificity for a triage test**				
BATF2	79·2 (66·5 - 88)	80	62·1 (50·2 - 72·7)	90·3 (83·5 - 94·5)
Kaforou25	79·2 (66·5 - 88)		62·1 (50·2 - 72·7)	90·3 (83·5 - 94·5)
Roe3	79·2 (66·5 - 88)		62·1 (50·2 - 72·7)	90·3 (83·5 - 94·5)
Sweeney3	88·7 (77·4 - 94·7)		64·7 (53·3 - 74·7)	94·5 (88·4 - 97·4)
**At minimum specificity for a confirmatory test**				
BATF2	41·5 (29·3 - 54·9)	98	89·6 (71·8 - 96·7)	80·2 (73·2 - 85·7)
Kaforou25	32·1 (21·1 - 45·5)		86·9 (65·9 - 95·8)	77·7 (70·7 - 83·4)
Roe3	34 (22·7 - 47·4)		87·5 (67·3 - 96)	78·2 (71·2 - 83·9)
Sweeney3	45·3 (32·7 - 58·5)		90·4 (73·6 - 96·9)	81·2 (74·3 - 86·6)
**At minimum sensitivity for a confirmatory test**				
BATF2	65	85·2 (78 - 90·3)	64·5 (51·1 - 75·9)	85·5 (78·3 - 90·5)
Kaforou25		91·4 (85·3 - 95·1)	75·8 (61·7 - 85·9)	86·3 (79·5 - 91·1)
Roe3		93 (87·2 - 96·3)	79·3 (65·1 - 88·7)	86·5 (79·8 - 91·2)
Sweeney3		93·8 (88·2 - 96·8)	81·2 (67 - 90·1)	86·6 (79·9 - 91·3)

Figure S9. Sensitivity analysis in ComBat batch-corrected gene expression data. A. Paired comparisons of area under the receiver operating characteristic curves (AUROCs) against the best-performing signature in this analysis (Roe3). **B.** Paired comparisons of ROC curves between HIV-infected and HIV-uninfected patients. Shaded areas represent the 95% confidence interval of the ROC curves. AUROC values are indicated with 95% confidence intervals in brackets. P values are derived from DeLong tests. **C.** ROC curves benchmarked against minimum (dashed black box) and optimum (blue box) target criteria for a TB triage test. Blue shaded area represents the 95% confidence interval of the ROC curve. AUROC values are indicated with 95% confidence intervals in brackets. **D.** Diagnostic performance metrics at different thresholds. CI = confidence interval; HIV = human immunodeficiency virus; NPV = negative predictive value; PPV = positive predictive value.

A.

Signature	AUROC, % (95% CI)	p-value
Roe3	86·6 (80·3 - 92·8)	ref
BATF2	85·6 (79·4 - 91·7)	0·41
Sweeney3	85 (78·5 - 91·5)	0·55
Kaforou25	85 (78·6 - 91·3)	0·43

B.

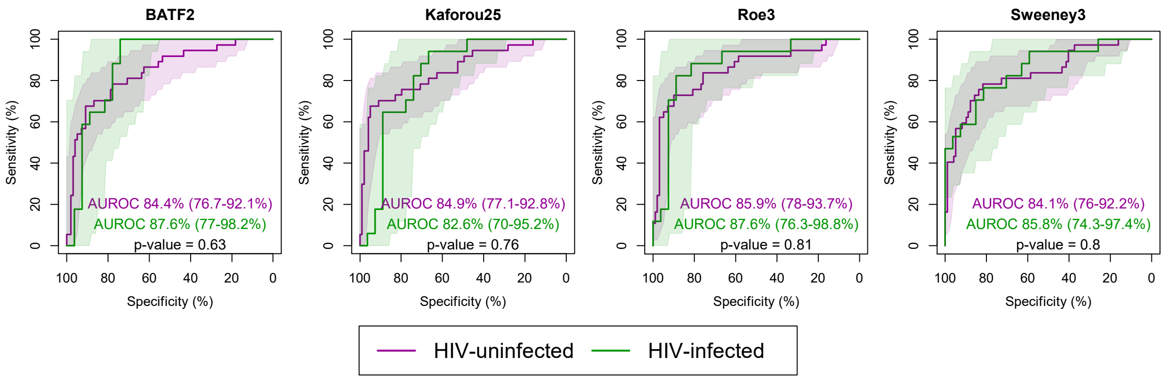

C.

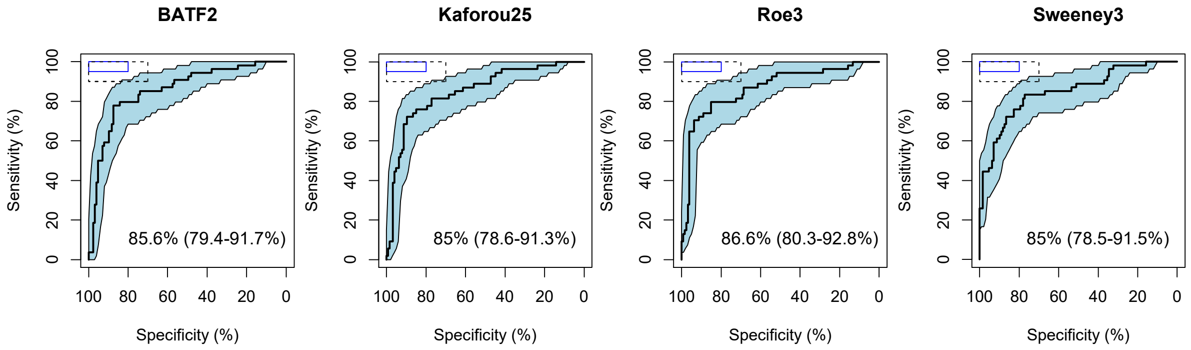

D.

	Sensitivity, % (95% CI)	Specificity, % (95% CI)	PPV, % (95% CI)	NPV, % (95% CI)
**At maximum Youden index**				
BATF2	77·8 (65·1 - 86·8)	87·4 (80·5 - 92·1)	72·4 (59·8 - 82·2)	90·2 (83·7 - 94·3)
Kaforou25	72·2 (59·1 - 82·4)	89·8 (83·3 - 93·9)	75 (61·8 - 84·8)	88·4 (81·7 - 92·8)
Roe3	79·6 (67·1 - 88·2)	85 (77·8 - 90·2)	69·4 (57 - 79·4)	90·8 (84·2 - 94·8)
Sweeney3	83·3 (71·3 - 91)	77·2 (69·1 - 83·6)	60·8 (49·4 - 71·1)	91·6 (84·8 - 95·5)
**At minimum sensitivity for a triage test**				
BATF2	90	56·7 (48 - 65)	46·9 (37·6 - 56·5)	93 (85·1 - 96·9)
Kaforou25		47·2 (38·8 - 55·9)	42 (33·4 - 51·2)	91·7 (82·5 - 96·3)
Roe3		56·7 (48 - 65)	46·9 (37·6 - 56·5)	93 (85·1 - 96·9)
Sweeney3		37 (29·1 - 45·7)	37·8 (29·9 - 46·4)	89·7 (78·6 - 95·4)
**At minimum specificity for a triage test**				
BATF2	85·2 (73·4 - 92·3)	70	54·7 (44·1 - 64·9)	91·7 (84·5 - 95·8)
Kaforou25	81·5 (69·2 - 89·6)		53·6 (42·9 - 64)	89·9 (82·4 - 94·4)
Roe3	81·5 (69·2 - 89·6)		53·6 (42·9 - 64)	89·9 (82·4 - 94·4)
Sweeney3	83·3 (71·3 - 91)		54·2 (43·5 - 64·4)	90·8 (83·4 - 95·1)
**At optimum sensitivity for a triage test**				
BATF2	95	37·8 (29·8 - 46·5)	39·4 (31·4 - 47·9)	94·7 (84·8 - 98·3)
Kaforou25		41·7 (33·5 - 50·4)	40·9 (32·7 - 49·7)	95·2 (86·1 - 98·4)
Roe3		28·3 (21·2 - 36·7)	36·1 (28·6 - 44·2)	93 (80·6 - 97·7)
Sweeney3		34·6 (26·9 - 43·3)	38·2 (30·4 - 46·6)	94·2 (83·6 - 98·1)
**At optimum specificity for a triage test**				
BATF2	79·6 (67·1 - 88·2)	80	62·9 (51 - 73·3)	90·2 (83·4 - 94·5)
Kaforou25	75·9 (63·1 - 85·4)		61·7 (49·7 - 72·5)	88·7 (81·6 - 93·2)
Roe3	79·6 (67·1 - 88·2)		62·9 (51 - 73·3)	90·2 (83·4 - 94·5)
Sweeney3	75·9 (63·1 - 85·4)		61·7 (49·7 - 72·5)	88·7 (81·6 - 93·2)
**At minimum specificity for a confirmatory test**				
BATF2	3·7 (1 - 12·5)	98	44·1 (13·1 - 80·5)	70·5 (63·4 - 76·8)
Kaforou25	9·3 (4 - 19·9)		66·3 (32·8 - 88·8)	71·8 (64·6 - 77·9)
Roe3	14·8 (7·7 - 26·6)		75·9 (45·8 - 92·1)	73 (65·9 - 79·1)
Sweeney3	44·4 (32 - 57·6)		90·4 (73·7 - 97)	80·6 (73·6 - 86)
**At minimum sensitivity for a confirmatory test**				
BATF2	65	88·2 (81·4 - 92·7)	70·1 (56·3 - 80·9)	85·6 (78·5 - 90·6)
Kaforou25		91·3 (85·2 - 95·1)	76·1 (62·1 - 86·1)	86 (79·1 - 90·9)
Roe3		93·7 (88·1 - 96·8)	81·4 (67·4 - 90·3)	86·3 (79·6 - 91·1)
Sweeney3		88·2 (81·4 - 92·7)	70·1 (56·3 - 80·9)	85·6 (78·5 - 90·6)
